# Ultrafast Thermal Imprinting of Plasmonic Hotspots

**DOI:** 10.1002/adma.202105192

**Published:** 2021-10-07

**Authors:** Sven H. C. Askes, Erik C. Garnett

**Affiliations:** ^1^ Center for Nanophotonics AMOLF Science Park 104 Amsterdam 1098 XG The Netherlands

**Keywords:** heterogeneous catalysis, nanoscale heat, photothermal, plasmonics, thermal hotspots, transition metal nitrides, ultrafast heating

## Abstract

Plasmonic photochemistry is driven by a rich collection of near‐field, hot charge carrier, energy transfer, and thermal effects, most often accomplished by continuous wave illumination. Heat generation is usually considered undesirable, because noble metal nanoparticles heat up isotropically, losing the extreme energy confinement of the optical resonance. Here it is demonstrated through optical and heat‐transfer modelling that the judicious choice of nanoreactor geometry and material enables the direct thermal imprint of plasmonic optical absorption hotspots onto the lattice with high fidelity. Transition metal nitrides (TMNs, e.g., TiN/HfN) embody the ideal material requirements, where ultrafast electron–phonon coupling prevents fast electronic heat dissipation and low thermal conductivity prolongs the heat confinement. The extreme energy confinement leads to unprecedented peak temperatures and internal heat gradients (>10 K nm^−1^) that cannot be achieved using noble metals or any current heating method. TMN nanoreactors consequently yield up to ten thousand times more product in pulsed photothermal chemical conversion compared with noble metals (Ag, Au, Cu). These findings open up a completely unexplored realm of nano‐photochemistry, where adjacent reaction centers experience substantially different temperatures for hundreds of picoseconds, long enough for bond breaking to occur.

## Graphical abstract

Plasmonic heating ordinarily leads to isotropic heating of the material. However, through nanoparticle shaping, pulsed illumination, and the right choice of material (e.g., HfN), plasmonic absorption hotspots can be directly imprinted on the lattice as heat. This approach results in extreme thermal hotspots, lasting hundreds of picoseconds, which can boost the chemical reactivity of catalytic nanoreactors by orders of magnitude.

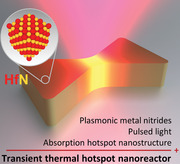

## 1 Introduction

Temperature is one of the most important parameters in chemical reactions, with a typical doubling of the rate with every 10 K increase. Catalyst selectivity, yield and product distribution is also often highly temperature dependent. Despite this immense practical importance, temperature has largely been controlled using the same approach for thousands of years: burning fuel to heat an entire reactor. In the last several decades, there has been a push to generate and control heat more locally using light, which allows for more rapid heating and cooling. This began with rapid thermal processing of silicon wafers for the semiconductor industry and more recently has been extended to the nanoscale with plasmonic heating, which is crucial for photothermal therapy^[^ ^1^, ^2^ ^]^ and may play an important role in plasmonic catalysis.^[^ ^3^ ^]^ Using optical excitation as a heat source affords wireless and remote spatiotemporal control over heat generation. For this purpose, plasmonic nanostructures are ideal light‐to‐heat converters, due to outstanding absorption cross sections, concentration of light in subwavelength volumes, and a highly tunable response across the visible and near infrared.^[^ ^4^, ^5^ ^]^ Plasmonic nanostructures have additionally attracted attention for driving chemistry due to the possibility of near‐field effects, hot carrier redox reactions, and a variety of energy transfer mechanisms.^[^ ^6^ ^]^

Squeezing the heat generation timeframe by using fs‐ to ns‐pulses greatly increases the attained peak temperature, temperature gradients, and the photochemical effects and yield in plasmonic photothermal catalysis and therapy;^[^ ^7^, ^8^ ^]^ the short bursts of photothermal work at much greater efficiency offset the photophysical idle‐time between pulses. Further squeezing heat generation in space adds an additional enhancement. In nanostructures with strongly localized optical hotspots, such as the star or diabolo geometry,^[^ ^9^, ^10^, ^11^, ^12^ ^]^ upon pulsed excitation, hot electrons are created and preferentially localized near the optical hotspot until electron–electron and electron–phonon scattering redistributes the energy isotropically.^[^ ^13^ ^]^ In the most well‐studied plasmonic materials (noble metals), direct conversion of the highly localized optical absorption to heat is prevented because electron–electron scattering and fast‐moving electrons^[^ ^14^ ^]^ quickly distribute energy in the nanostructure (<100 fs) before electron–phonon coupling occurs (0.1–10 ps) and thus before energy is transferred to the lattice and its slow‐moving phonons.^[^ ^6^, ^15^, ^16^ ^]^ After electron–phonon equilibration, a high overall thermal conductivity (κ > 300 W (m K)^−1^) further rapidly reduces any small existing thermal gradients. Therefore, a relatively low electron–phonon coupling coefficient (e.g., for Au: *G* = 2.8 × 10^16^ W m^−3^ K^−1^)^[^ ^17^ ^]^ and high thermal conductivity cause heat generation in noble metal nanostructures to be rather isotropic with weak localization. These factors combined make it difficult to reach high surface lattice temperatures (>500 K) and internal gradients (>10 K nm^−1^) by photothermal heating of metal nanostructures, especially for times long enough to break chemical bonds of surface‐adsorbed molecules (>1 ps). Existing solutions for this issue include antenna‐reactor complexes^[^ ^18^ ^]^ and plasmonic Fano effect heaters,^[^ ^19^ ^]^ which are both of intricate design and therefore challenging to fabricate. Additionally, even with modest heating noble metal nanostructures are prone to reshaping and fragmentation due to severe melting‐point depression of surface material, thereby undesirably altering their optical and physical properties during operation.^[^ ^20^, ^21^, ^22^, ^23^ ^]^

Here we show it is possible to directly transfer plasmonic optical hot spots to transient thermal hotspots with extreme peak temperatures and gradients by maximizing the rate of electron–phonon coupling and minimizing the overall thermal conductivity. Using optical and heat‐transfer modelling, we demonstrate that individual HfN nanoreactors, based on the diabolo geometry, fulfill these requirements and support long‐lasting transient thermal hotspots with extreme nanoscale temperature localization under pulsed illumination. We furthermore show that these transient thermal hotspots can be effectively utilized for driving a thermally activated chemical reaction. We benchmark our results against the performance of Au, and systematically investigate in which wavelength and particle size regime and under which laser excitation conditions HfN performs better than Au. Finally, we map out the performance of a range of other plasmonic metals and identify the influence of *G* and κ as main contributors for efficient transient thermal hotspots.

## 2 Results and Discussion

**Figure 1:**
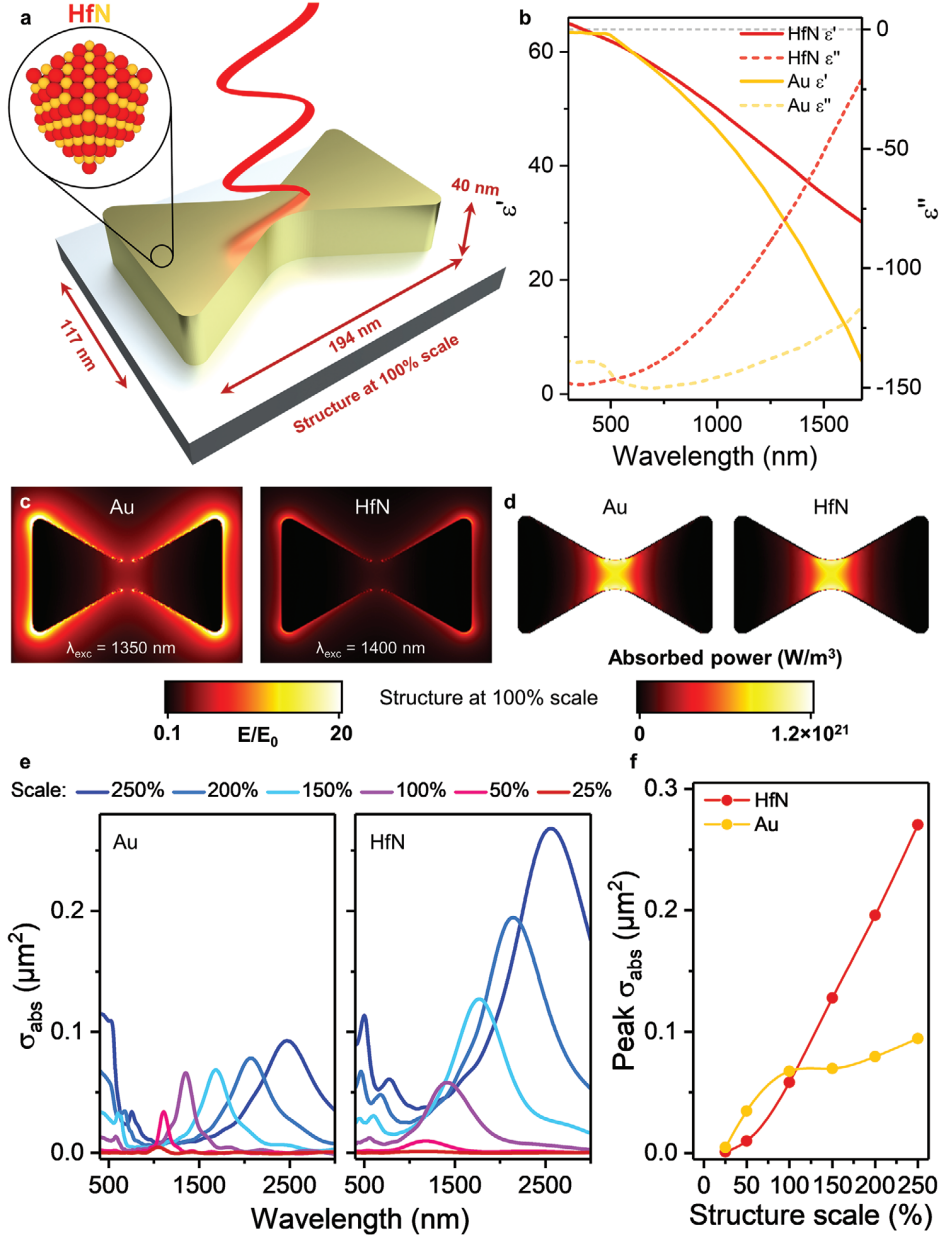
Optical characterization of HfN and Au diabolo nanoreactors on Al_2_O_3_ substrate in air. a) Dimensions of a HfN diabolo nanoreactor at 100% scale: length = 194 nm, width = 117 nm, width at waist = 28 nm, and height = 40 nm. b) Real (ε′, solid) and imaginary (ε′′, dashed) permittivity of thin film HfN^[^ ^28^ ^]^ and Au.^[^ ^35^ ^]^ c,d) Electric field (*E*/*E*_0_) (c) and absorbed power maps (*P*_abs_ ∝ −0.5ω|*E*/*E*_0_|^2^ε′′) (d) for the nanoreactor at 100% scale, at the top surface. Maps for different sizes shown in Figure S1 in the Supporting Information. e) Absorption cross section spectra at 25–250% scale. f) Peak absorption cross section as a function of structure scale. Scatter cross section spectra are shown Figure S2 in the Supporting Information.

The group 4 transition metal nitrides (TMNs), i.e., TiN, ZrN, and HfN are a relatively new class of plasmonic materials that embody the two requirements of low thermal conductivity and high electron phonon coupling.^[^ ^24^, ^25^, ^26^, ^27^, ^28^, ^29^ ^]^ Although each of these refractory materials has relatively low thermal conductivity (κ ≈ 20 W m^−1^ K^−1^)^[^ ^30^ ^]^ and high *G* (≥10^18^), we focus here on HfN because it has the highest melting point (*T*_m_ > 3300 °C),^[^ ^30^, ^31^ ^]^ high chemical resistance,^[^ ^30^ ^]^ presence of a catalytically active surface,^[^ ^32^ ^]^ and a high LSPR quality in the vis–NIR range.^[^ ^26^ ^]^ These properties make HfN attractive for photothermal applications under demanding conditions, such as photothermal catalysis. Furthermore, the optical constants of HfN can be tuned to a favorable trade‐off between metallic character (negative real permittivity ε′) and high optical losses (imaginary permittivity ε′′, **Figure** **1**b), leading to efficient light‐induced absorption and heating.^[^ ^26^, ^28^, ^33^, ^34^ ^]^

As photothermal nanoreactor in a heterogeneous gas‐phase environment we selected a diabolo geometry on Al_2_O_3_ substrate in air, and used finite‐difference time‐domain (FDTD) simulations to extract the electric field and absorbed power map for the structure made from Au or HfN at 25–250% scale (see Figure 1a for the dimensions at 100% scale). At each scale and for each material the same LSPR mode applies, peaking in the near‐infrared region, red‐shifting with larger size, red‐shifting slightly for HfN, localized externally on the four corners, and localized internally at the waist of the structure (Figure 1; Figures S1 and S2¸ Supporting Information). The majority of absorption occurs at the waist (>40× than on the corners), representing an absorption “hotspot.” Although HfN features lower plasmonic quality and lower electric field enhancements than Au, the higher optical losses in the material results in higher absorbed power for structures larger than ≈110% scale. For larger Au structures, the absorption flattens at the expense of higher scattering (Figure S2, Supporting Information). We reproduced the FDTD simulations for the diabolos free‐floating in water, and qualitatively found the same results (Figures S3 and S4, Supporting Information). At 100% scale, the absorption cross section for both materials was nearly equal (σ_abs_ = 0.0584 and 0.0676 µm^2^ for HfN and Au, respectively), which was therefore chosen as the starting point for a fair comparison of light‐to‐heat conversion with both materials.

**Figure 2:**
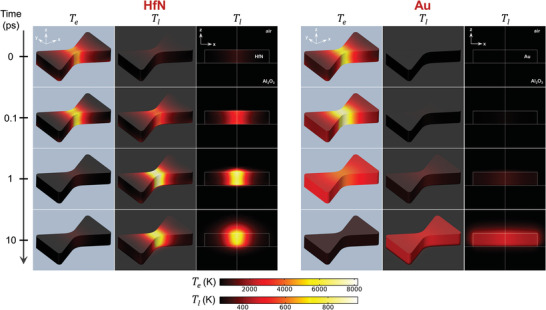
Evolution of electronic and lattice temperature during 50 fs pulsed excitation at 0.32 mJ cm^−2^ pulse energy (0.8 W cm^−2^) for nanoreactors at 100% scale (194 nm long, 117 nm wide, 40 nm high, 28 nm wide at waist). The full simulation is shown in Video S1 in the Supporting Information.

**Figure 3:**
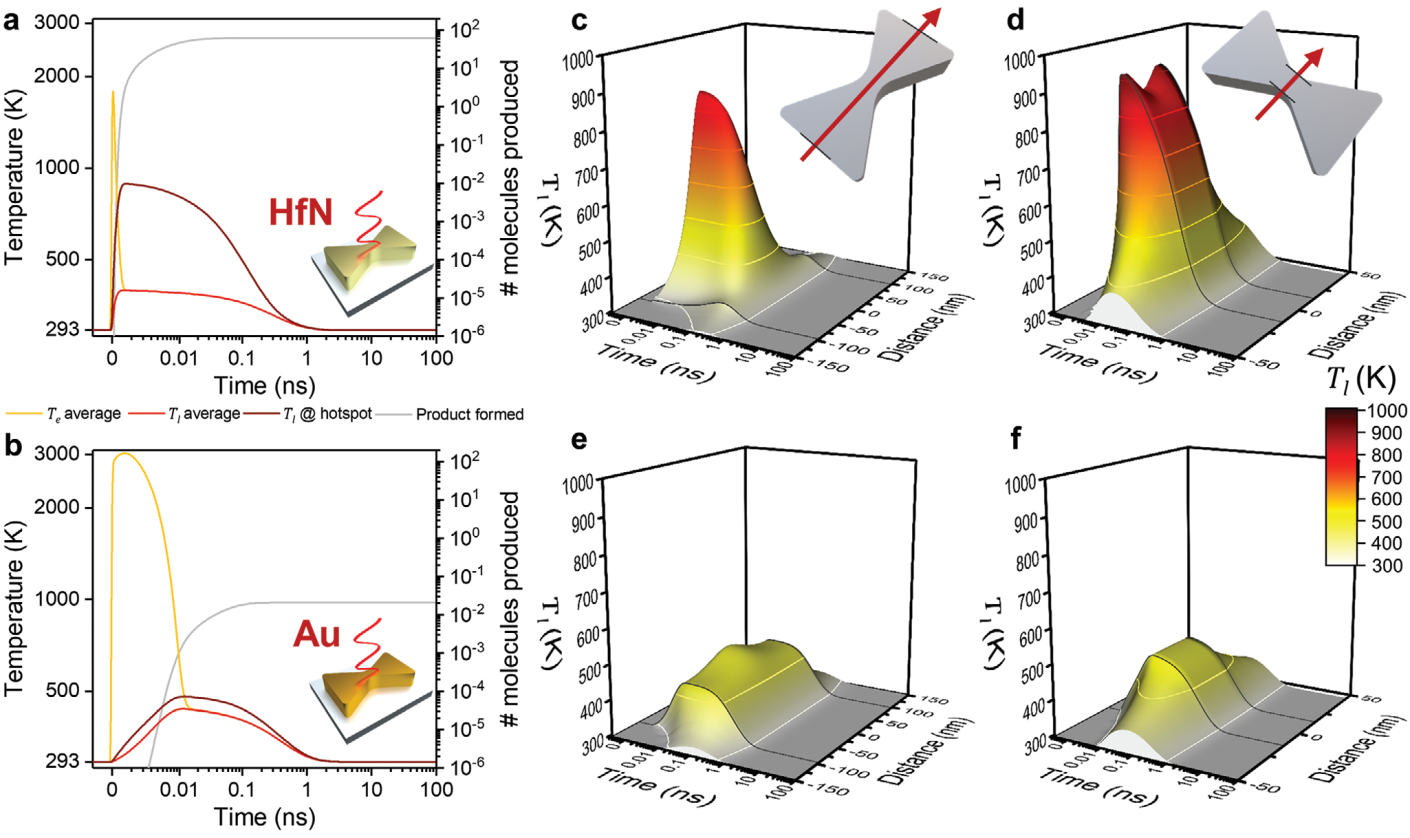
Evolution of electronic and lattice temperature for HfN and Au nanoreactors at 100% scale. a,b) Time‐evolution of volume‐averaged electronic (yellow) and lattice temperatures (red), the lattice temperature at the absorption hotspot (dark red), and the number of product molecules produced per pulse (grey) during 50 fs pulsed excitation at 0.32 mJ cm^−2^ pulse energy (0.8 W cm^−2^) for HfN (a) and Au (b) nanoreactors. c–f) Surface lattice temperature time‐evolution along the long (c,e) and short diabolo axis (d,f), as indicated by the red arrows, for HfN (c,d) and Au (e,f). Structure edges are indicated with black lines.

To examine the spatiotemporal evolution of temperature upon light excitation, the absorbed power maps were used as an electronic heat source input for finite element method simulations using a 3D two‐temperature model. In this model, electronic and lattice temperatures are separated (*T*_e_ and *T*_l_, respectively) and exchange energy proportional to the electron–phonon coupling coefficient (*G*) (see supporting information for a detailed description of the model). Collective heating was omitted from the model, i.e., the outer simulation boundaries were kept constant at 293 K. Notably, *G* for HfN is ≈50 × higher than that of Au, predicting a much faster heat exchange between electrons and the lattice. **Figures** **2** and **3**a,b shows the evolution of *T*_e_ and *T*_l_ after 50 fs pulsed excitation at low pulse intensity (0.32 mJ cm^−2^, 0.8 W cm^−2^). As expected for noble metals, for Au the electron–phonon coupling takes about 15 ps to reach full equilibrium.^[^ ^36^ ^]^ Within this timeframe, due to the large thermal conductivity of the electrons in Au, the electronic heat is distributed around the nanoparticle geometry and the sharp energy localization at the optical hotspot is lost. In contrast, complete electron–phonon equilibration in HfN only takes 0.5 ps, which prevents electronic heat distribution. As a consequence, the difference in peak surface lattice temperature after e‐ph equilibration is striking: whereas the optical hotspot in HfN reaches a peak temperature of 920 K, it is only 480 K for Au. For HfN, lattice heat is localized as an almost exact copy of the absorbed power map, while for Au, the imprint is almost completely lost. Furthermore, due to the low thermal conductivity, HfN maintains a high temperature at the optical hotspot for ≈400 ps, even though the internal thermal gradient across the structure is very high (16 K nm^−1^ in the first 10 ps). For both materials, thermal dissipation to the surroundings and substrate is complete within 2 ns. Overall, these results demonstrate that in pulsed‐excited HfN nanostructures optical hotspots are transformed to thermal hotspots with high fidelity, which gives rise to greater peak lattice temperatures on the nanoparticle surface and greater internal thermal gradients than those accomplished with Au.

An alternative strategy to confine heat in a small volume could be to simply use a much smaller nanostructure with a size comparable to the diabolo absorption hotspot, and thereby miniaturize the heat source. However, the loss of absorption cross section is detrimental for the light‐to‐heat conversion efficiency of the reactor: upon removal of the diabolo wings, the remaining Au nanorod “core” reaches only 320 K under identical illumination due to the ≈100× lower maximum absorption cross section (Figure S7, Supporting Information). Likewise, we recently demonstrated with the same simulation framework that at 4× higher pulse energy (3.2 W cm^−2^), a 20 nm Au nanosphere with the approximate size of the nanodiabolo constriction (at 100% scale) reaches an averaged peak lattice temperature of 530 K in H_2_O.^[^ ^24^ ^]^ These peak temperatures are in stark contrast with the 920 K transiently reached by the HfN nanoreactor. Evidently, even though heat is selectively generated at the optical hotspot, the rest of the diabolo reactor has a crucial role as antenna.

To show that these results have profound implications for photothermal catalysis, an arbitrary thermally‐activated first‐order chemical reaction was executed on the nanoreactor surface (Reactant → Product), where the conversion rate was governed by a temperature‐adjusted Arrhenius equation with 1 eV activation energy. Details, further considerations, and the accuracy of this model are discussed in Section S3.4 in the Supporting Information. Due to the exponential dependence on surface temperature, even though the reaction takes place in a smaller area at the transient thermal hotspot, more than three orders of magnitude higher reaction yields per pulse were calculated for HfN than for Au (Figure 3a,b). Furthermore, comparing these results with continuous wave (CW) illumination, which increases the temperature by a mere 0.1 mK under such low light intensity (0.8 W cm^−2^), the HfN nanoreactor yielded 6 orders of magnitude more photoproduct versus 2–3 orders of magnitude for the Au nanoreactor (Figure S8, Supporting Information). These initial results clearly demonstrated the potential of spatiotemporal heat confinement using plasmonic transient thermal hotspots.

**Figure 4:**
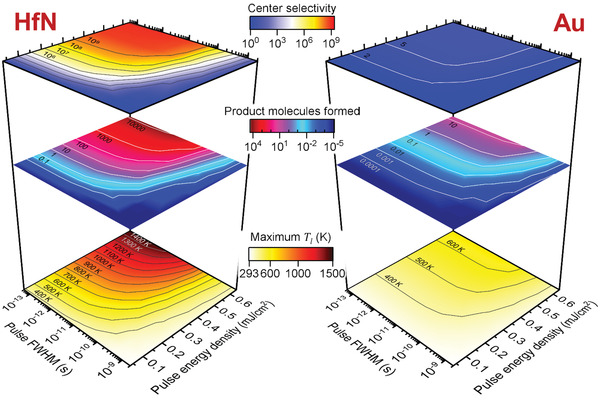
Influence of laser pulse duration (Gaussian time profile, FWHM = full width at half maximum) and pulse energy density on the maximum attained lattice temperature (bottom), amount of product molecules formed per pulse (middle), and the center selectivity of the chemical product formation (top) for HfN (left) and Au (right) nanoreactors at 100% scale. A total of 48 simulations were used to obtain the contour plots. The center selectivity is defined as the ratio of average product formed at the waistline and the average product formed at the outer edge. Reactant depletion does not occur: the maximum reactant conversion is 60–67% for HfN at 0.64 mJ cm^−2^ (1.6 W cm^−2^) and ≤4 ps FWHM.

The optical excitation parameters profoundly influence the temperature dynamics, which consequentially affect the product formation quantity and location (**Figure** **4**). We systematically varied laser pulse duration and energy density, and evaluated the maximum *T*_l_, number of product molecules formed, and the spatial selectivity of the chemical reaction for the optical hotspot. For both HfN and Au, an increase in pulse energy translated to a larger peak lattice temperature on the surface, but the slope was more than triple for HfN because a smaller volume is heated (1.9 K vs 0.6 K increase per µJ cm^−2^ increase for HfN and Au, respectively, at 50 fs pulse duration). Although we do not consider melting in our model, it must be noted that the sharp features of the Au nanoreactor surface may deform at temperatures as low as 400 K due to melting point depression,^[^ ^22^, ^23^ ^]^ whereas TMNs resist deformation.^[^ ^37^ ^]^ Meanwhile, for both materials a shortening of the pulse duration to the fs‐regime leads to a dramatic increase in peak lattice temperature on the surface, representing a transition to a regime where the transient thermal hotspot is most intense. An important observation is that most of the enhancement is gained by shortening the pulse from 2 ns to 10 ps, and the enhancement flattens off for further shortening to the fs‐regime. This occurs because the pulse duration needs to be much shorter than the internal thermal equilibration time of the nanoreactor, which will also vary with nanoreactor geometry, size, and environment (see also results for a nanosphere dimer configuration, below). Importantly, the ps‐pulse regime at visible and near‐infrared wavelengths is nowadays easily accessible using affordable and high‐power supercontinuum lasers. Further, the product yield and center selectivity follow the trends in *T*_l_: shorter and more intense pulses lead to more product formed with higher center selectivity, where the HfN nanoreactor produces ≈1000× more product than Au across the parameter space. Notably, the intense and long‐lived transient thermal hotspot in the HfN nanoreactor results in a center selectivity as high as 10^9^, compared to maximally 7 in Au. These results demonstrate that laser pulse duration and energy are handles to tune the temperature response and thermocatalytic performance of plasmonic nanoreactors, where HfN clearly yields more product with higher spatial selectivity than Au, under any pulsed irradiation condition.

Next, we varied nanoreactor scale from 25–250%, realizing that σ_abs_ is no longer approximately equal (Figure 1), and examined temperature dynamics and chemical conversion (discussed in detail in Section S4.4., Figures S9–S13, and Videos S1 and S2, Supporting Information). The results illustrated the limits of the transient thermal hotspot strategy: only when the Au nanoreactor absorbs more than twice as much light (at <≈75% scale), isotropic heating yields more product. Finetuning of the optical constants of the TMN is therefore key for maximizing the nanoreactor absorbance for specific wavelength regimes.^[^ ^26^, ^28^ ^]^ Further, HfN nanoreactors at any scale exhibited enormous transient surface temperature gradients (3.9–43 K nm^−1^), which can be exploited in nanoscale photo‐thermoelectric devices, could prove useful in heat assisted magnetic recording, and would have completely unknown effects in chemical reactions. For instance, the ultrafast heating and cooling down of adsorbates at transient thermal hotspots enables the initiation of catalytic cycles and the analysis of kinetically trapped intermediates using pump‐probe spectroscopy. Finally, at any scale the HfN nanoreactor robustly exhibited extreme product localization at the optical hotspot (>10^5^), which can be leveraged in nanofabrication strategies.

**Figure 5:**
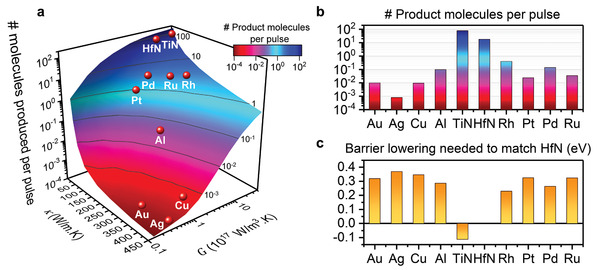
Influence of material properties on the chemical product formation of nanoreactors at 100% scale. a) Influence of overall thermal conductivity (κ) and electron–phonon coupling constant (*G*), while keeping the absorption cross section and all material properties at those for HfN. Estimated location of other plasmonic metals, based on their κ and *G* values, are shown as red spheres. A total of 72 simulations were used to obtain the plot. b) Product formation of nanoreactors at 100% scale made from ten different plasmonic metals, fully accounting for absorption cross section, 3D absorption profile, and material properties. c) Activation energy lowering needed for other metals to match the performance of HfN nanoreactors. For calculation, see Figure S17 in the Supporting Information. All simulations performed with 50 fs laser pulse at 0.32 mJ cm^−2^ (0.8 W cm^−2^).

We systematically analyzed the influence of two crucial material properties in this strategy: the electron–phonon coupling constant *G* and the overall thermal conductivity κ (**Figure** **5**). For direct comparison, the nanoreactor was scaled at 100% and the absorption cross section and other material properties were kept at those of HfN. The data show that the most chemical product is formed for metals with very low κ and very high *G*, with an additive effect of both variables. The group 4b TMNs fulfill these criteria outstandingly, with *G* = 10–50 × 10^17^ W m^−3^ K^−1^ and κ ≈ 20 W m^−1^ K^−1^.^[^ ^27^, ^38^ ^]^ In contrast, parameter values that correspond to the noble metals Au, Ag, and Cu (*G* ≤ 1 × 10^17^ W m^−3^ k^−1^ and κ > 300 W m^−1^ K^−1^) resulted in 4–5 orders of magnitude lower chemical conversion. Interestingly, we also identified a region of plasmonic materials with intermediate properties, such as Rh (*G* = 19.5 × 10^17^ W m^−3^ K^−1^ and κ = 150 W m^−1^ K^−1^) and Pd (*G* = 5 × 10^17^ W m^−3^ K^−1^ and κ = 71 W m^−1^ K^−1^), where photothermal conversion was only 10–100× lower than with the TMNs. At the same time, these metals are important catalytic materials that are widely used in chemical industry.

To validate the predictions, the simulations were repeated for a wide range of plasmonic metals, including Ag, Cu, Al, TiN, Rh, Pt, Pd, and Ru, where optical absorption profile, cross section, and all material properties were taken into consideration (Figure 5b; Figures S14–S16, Supporting Information). The product formation narrowly followed the predictions from strictly varying *G* and κ, with deviations from the trend directly correlated with differences in absorption cross section. Thus, other material properties influence transient thermal hotspot formation to a lower degree. Due to the higher absorption, high quality TiN performs even better than HfN in this wavelength range, although the simulations are sensitive to the dielectric function used, which varies substantially for TMNs.^[^ ^26^ ^]^ Rh and Pd are the best of the rest, with at least one order of magnitude higher performance than noble metals. Due to their relatively low absorption cross section, Pd, Ru, Rh, and Pt nanoreactors perform worse than expected based on their *G* and κ values, but nanophotonic strategies to boost their absorption have been demonstrated before.^[^ ^39^ ^]^

In reality, the magnitude of the catalytic activation barrier for adsorbates varies strongly from metal to metal, making our comparison between different metals unfair. To address this point, we examined the activation energy lowering required for each metal to achieve the same product yield as HfN (Figure 5c). For instance for Cu, the activation barrier needs to be lowered from 1 to 0.64 eV to reach the same product yield. All of the tested metals require barrier lowering of at least 0.24 eV to match the photocatalytic conversion of HfN. Thus, other metals need to be substantially more catalytically active than HfN for a given thermal reaction to compete with TMNs under the same illumination and geometrical conditions. The transient thermal hotspot strategy thus represents a brute‐force approach for thermally activated chemistry and enhances efficiency without the requirement for a good catalytic surface; adsorption of reactants at the optical hotspot is enough to drive thermal activation. Although TMN surfaces may be catalytically active in itself,^[^ ^32^, ^40^ ^]^ they are thus far poorly explored. Naturally, for a given catalytic reaction, the TMN surface could be modified with cocatalyst nanoparticles to achieve the best of both worlds: catalytically active surface and efficient transient thermal hotspots. Another possibility is to explore ternary metal nitrides as plasmonic photothermal catalysts, which have recently been theoretically mapped and assessed for stability.^[^ ^41^ ^]^

**Figure 6:**
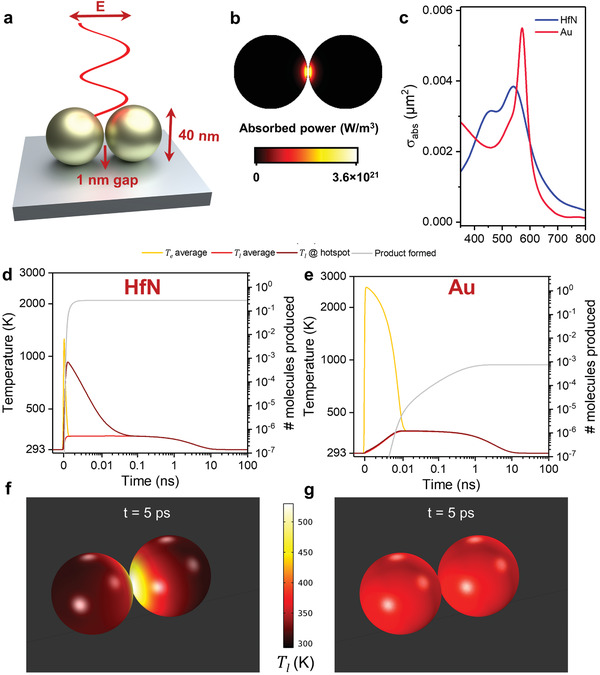
Optical and heat transfer simulations for nanosphere dimer nanoreactors with visible light absorption. a) Dimensions of the nanosphere dimer. b) Absorbed power map for the HfN dimer at half height. c) Absorption cross section spectra for HfN and Au nanosphere dimers. d,e) Time‐evolution of volume‐averaged electronic (yellow) and lattice temperatures (red), the lattice temperature at the absorption hotspot (dark red), and the number of product molecules produced per pulse (grey) during 50 fs pulsed excitation at 0.32 mJ cm^−2^ pulse energy (0.8 W cm^−2^) for HfN (d, λ_exc_ = 542 nm) and Au (e, λ_exc_ = 571 nm) nanosphere dimer reactors. f,g) Lattice temperatures at *t* = 5 ps for HfN (f) and Au (g) nanoreactors. The full simulation is shown in Video S3 in the Supporting Information.

Finally, to extend our findings to other nanophotonic configurations and demonstrate that transient thermal hotspots are just as readily achieved using visible light excitation, the simulations were carried out for a nanosphere dimer nanoreactor (40 nm diameter, 1 nm spacing, **Figure** **6**). This configuration features a coupled resonance at 570 and 540 nm for Au and HfN (Figure 6a–c; Figure S18, Supporting Information), respectively, while the absorption is greatly localized in the material surrounding the gap (>480× than on the outer edge). At resonance, the absorption cross section for Au is 46% higher than for HfN (σ_abs_ = 561 and 385 nm^2^, respectively, Figure 6c). The spatiotemporal evolution of temperature upon light excitation tells a similar story as for small nanodiabolos: whereas for HfN the absorption hotspot is thermally imprinted during the first 100 fs after the pulse, for Au the fast electronic heat dissipation causes completely isotropic heat generation (Figure 6d–g). Consequently, the HfN nanoreactor reaches a peak surface lattice temperature of 920 K (390 K for Au), yields >200× more product for <1 ps pulse width, and allows highly selective product formation at the optical hotspot (for a full pulse width and energy density sweep see Figure S19 in the Supporting Information). Thus, even for simple systems that spontaneously form upon colloidal aggregation or deposition, and for those that are excited at visible wavelengths, our results suggest that transient thermal hotspots can be exploited to accomplish higher photothermal light‐to‐product yields.

## 3 Conclusions

With this work we have introduced the concept of direct lattice heating to create plasmonic transient thermal hotspots, and we have shown that the most intense and long‐lived transient thermal hotspots can be achieved through a combination of (1) a plasmonic nanoreactor geometry with a strong absorption hotspot, (2) fs‐ to ps‐pulsed laser excitation, (3) ultrafast electron–phonon coupling, and (4) low thermal conductivity. TMNs embody these unique requirements and perform orders of magnitude better than noble metals for pulsed photothermal catalysis, based on brute‐force thermal conversion. At the same time, their refractory properties ensure the robustness of the nanoreactor, with resistance to melting and deformation. The strategy holds up at any nanoreactor size, at any vis–NIR wavelength, and is already effective with low‐intensity and cost‐effective ps‐pulsed excitation. We envision that TMN nanoparticle assemblies can be used at the heart of a room‐temperature flow‐cell reactor, powered by a supercontinuum laser, powered in turn by sustainable electricity, where thermally activated chemistry is performed that ordinarily requires high temperature reactors. Although we focused primarily on the diabolo geometry, which can be challenging to fabricate from TMNs at small scale,^[^ ^28^ ^]^ the choice in reactor geometries with absorption hotspots across the visible and NIR wavelengths is bountiful. Low‐tech, cost‐efficient, and scale‐up ready designs are presently available,^[^ ^42^ ^]^ such as plasmonic metamaterial absorbers containing particles on a mirror.^[^ ^43^, ^44^ ^]^ Our findings also apply to designing safer and more efficient photothermal therapy, where less irradiation dose is required to reach the same therapeutic effect. Finally, the high and long‐lasting thermal gradients associated with transient thermal hotspots open up an entirely unexplored realm of chemistry where adjacent reactive sites are held at substantially different temperatures. These extreme temperature gradients may also prove useful in nanoscale photo‐thermoelectric devices and heat‐assisted magnetic recording.

## Conflict of Interest

The authors declare no conflict of interest.

## Supporting information

Supporting Information

Supplemental Video 1

Supplemental Video 2

Supplemental Video 3

## Data Availability

Research data are not shared.
