# Supplementary material for: Ultrafast Thermal Imprinting of Plasmonic Hotspots
Source: Adv Mater. 2021 Oct 7;33(49):2105192. doi: 10.1002/adma.202105192 (PMC11468741; doi:10.1002/adma.202105192)
Supplement: Supplementary file 1 — Supporting Information [file ADMA-33-2105192-s004.pdf]

© 2021 Wiley-VCH GmbH

ADVANCED MATERIALS

Supporting Information

for *Adv. Mater.,* DOI: 10.1002/adma.202105192

Ultrafast Thermal Imprinting of Plasmonic Hotspots

*Sven H. C. Askes and Erik C. Garnett**

Ultrafast thermal imprinting of plasmonic hotspots

Sven H.C. Askes and Erik C. Garnett

Supporting information

1. Optical properties of HfN and Au diabolos on Al_2O_3 in air 2
2. Optical properties of HfN and Au diabolos in water 4
3. Methodology of three-dimensional heat transfer simulations 6
   3.1. Description of COMSOL geometry and simulation settings 6
   3.2. Heat transfer and electron-phonon coupling 7
   3.3. Optical heat source 14
   3.4. Thermally activated chemical reaction 16
4. Two-temperature heat transfer modelling 19
   4.1. Au nanorod on Al_2O_3 in air 19
   4.2. HfN and Au diabolo on Al_2O_3 in air 20
   4.3. HfN and Au diabolo in H_2O 25
   4.4. Discussion on the effect of nanoreactor scale 28
5. FDTD and heat transfer modelling for other metals 31
6. FDTD and heat transfer modelling for a nanosphere dimer 36
7. References 38

1. Optical properties of HfN and Au diabolos on Al_2O_3 in air

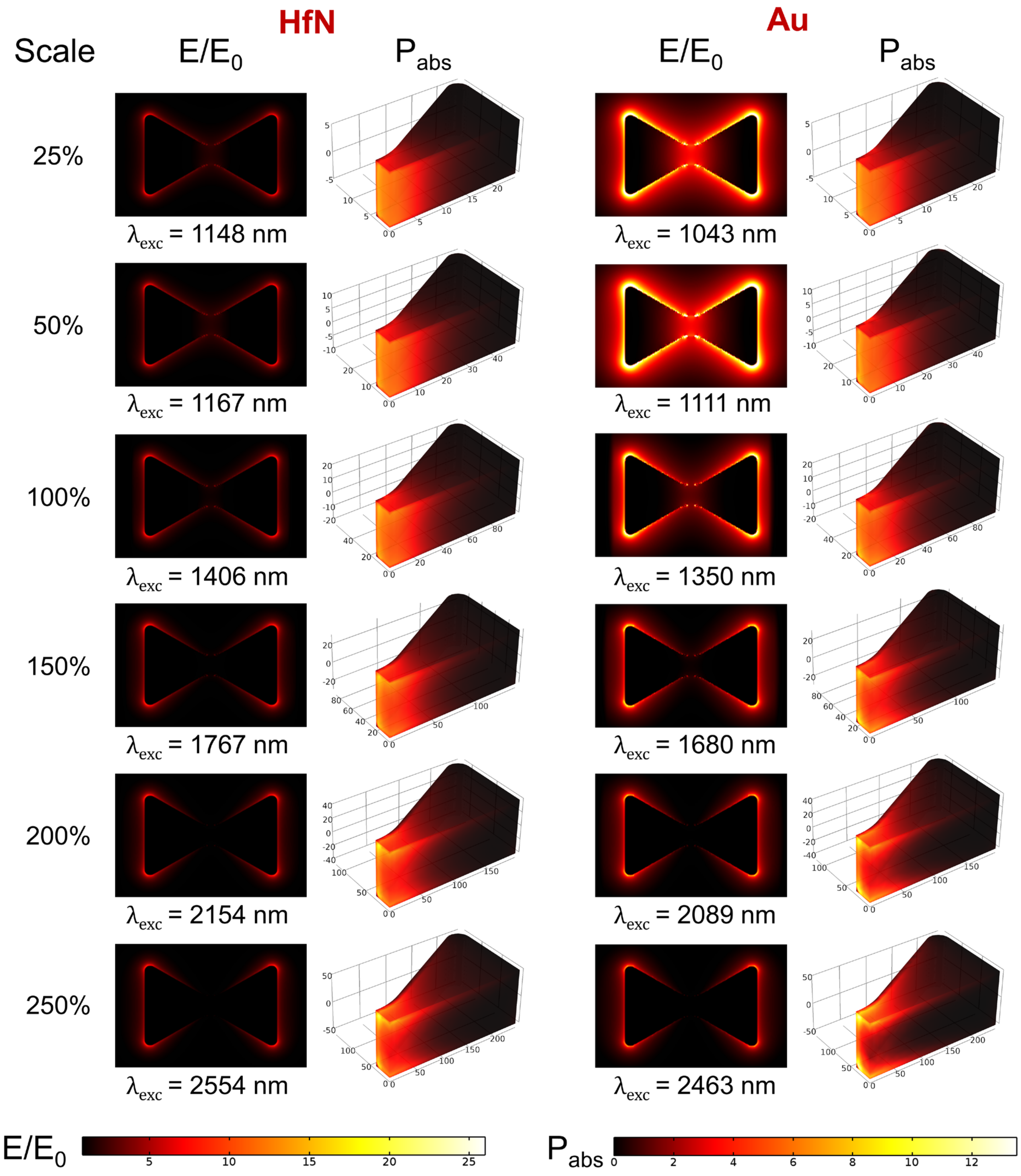

Figure S1. FDTD-simulated electric field maps (at top of structure) and normalized spatial absorbed power maps in HfN (left two columns) and Au diabolos (right two columns) on Al_2O_3 substrate and in air surroundings for 25 – 250% scaled structures. Simulations were performed with plane wave excitation polarized along the long axis of the diabolo and at their respective peak absorption wavelength, as designated underneath each E-field map. Axes show dimensions in nm.

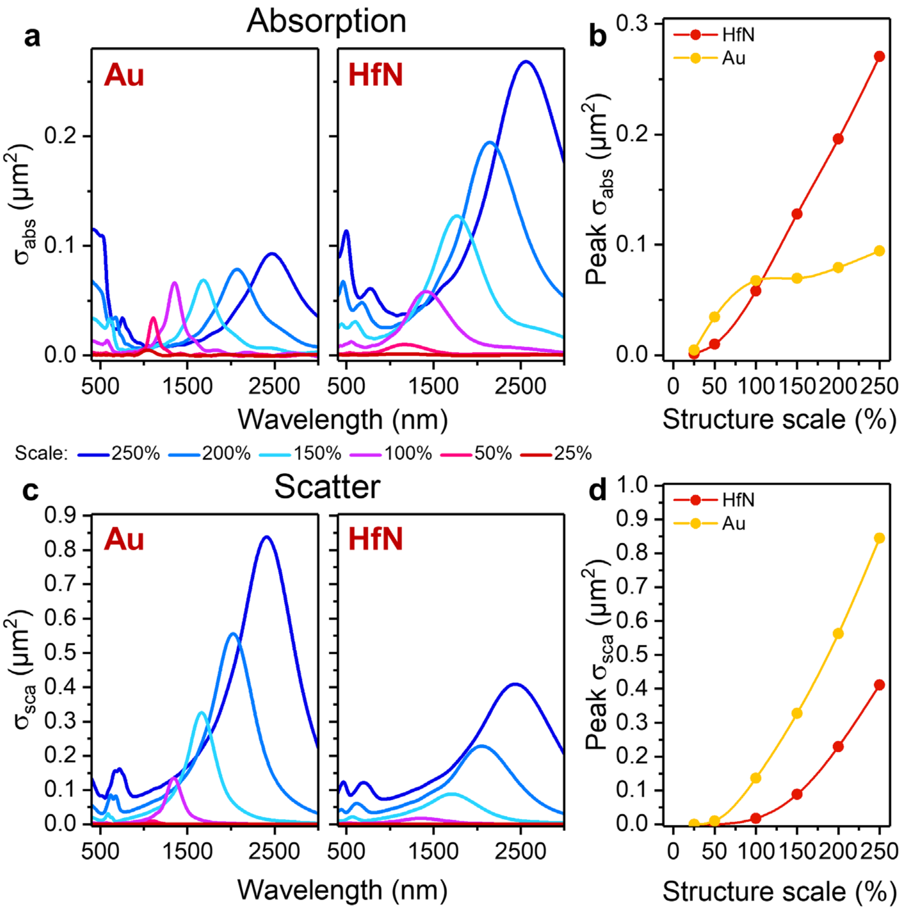

Figure S2. Summary of FDTD-simulated optical properties of HfN and Au diabolos on Al_2O_3 substrate and in air surroundings. Simulations were performed with plane wave excitation polarized along the long axis of the diabolo. a&c) Absorption and scatter cross section spectra for Au (left) and HfN (right) for 25 – 250% scaled structures. b&d) Peak absorption (b) and scatter cross sections (d) as a function of structure scaling.

2. Optical properties of HfN and Au diabolos in water

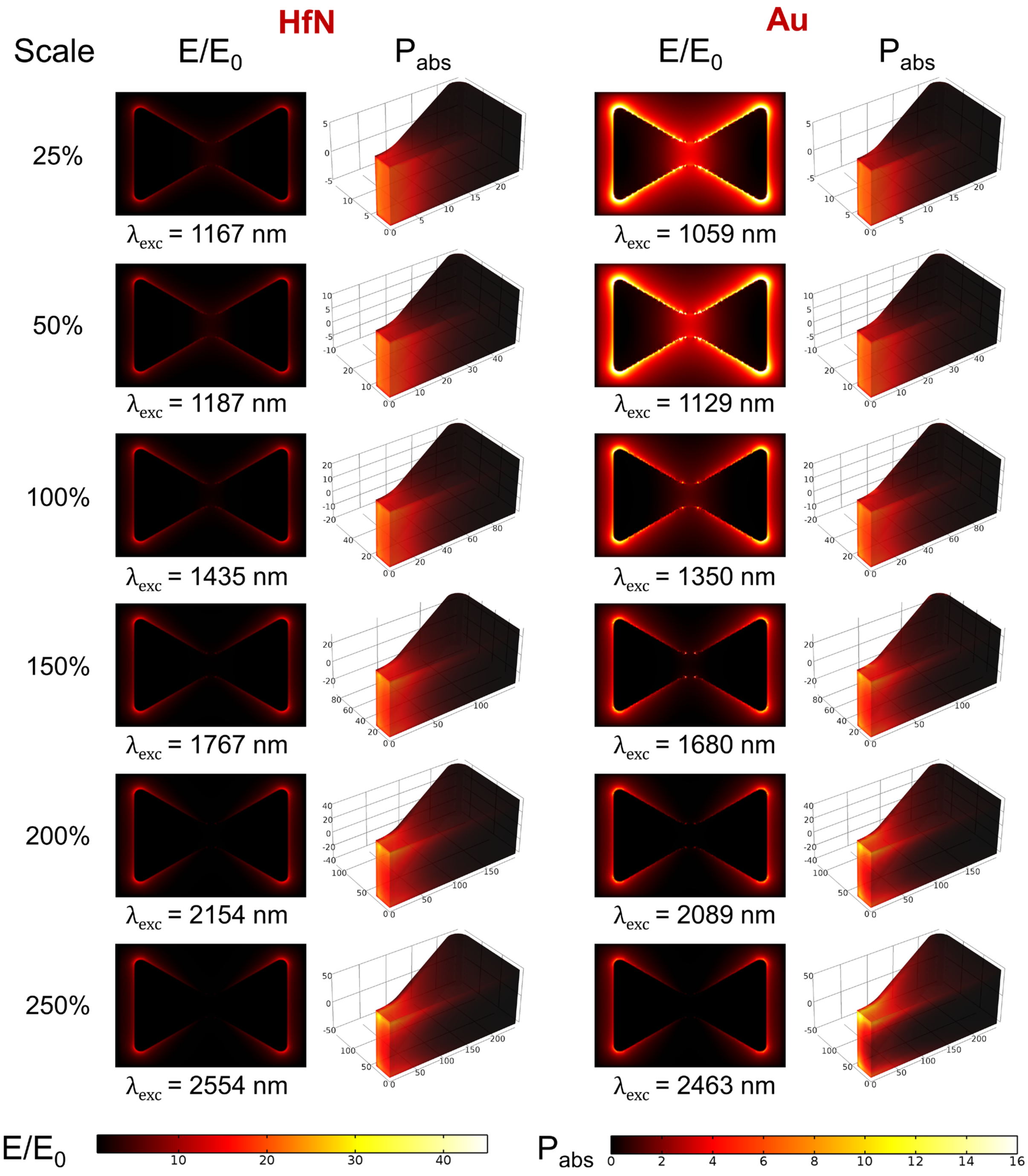

Figure S3. FDTD-simulated electric field maps (at top of structure) and normalized spatial absorbed power maps in HfN (left two columns) and Au diabolos (right two columns) in water for 25 – 250% scaled structures. Simulations were performed with plane wave excitation polarized along the long axis of the diabolo and at their respective peak absorption wavelength, as designated underneath each E-field map. Axes show dimensions in nm.

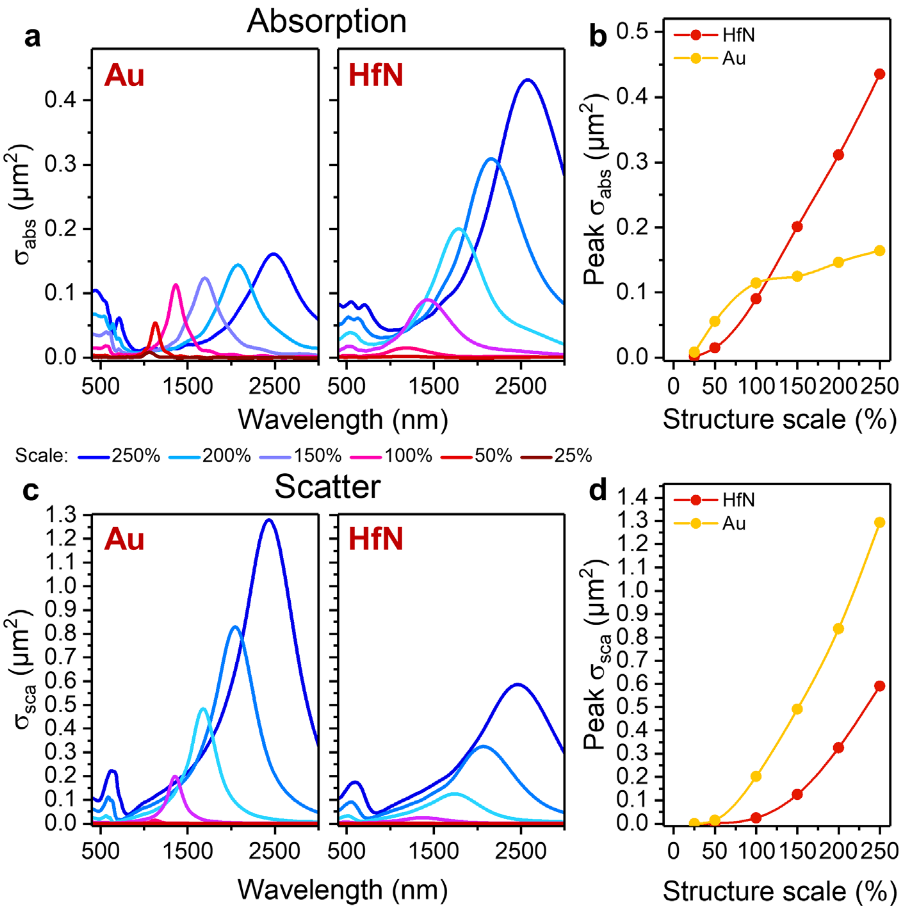

Figure S4. Summary of FDTD-simulated optical properties of HfN and Au diabolos suspended in water. Simulations were performed with plane wave excitation polarized along the long axis of the diabolo. a&c) Absorption and scatter cross section spectra for Au (left) and HfN (right) for 25 – 250% scaled structures. b&d) Peak absorption (b) and scatter cross sections (d) as a function of structure scaling.

3. Methodology of three-dimensional heat transfer simulations

3.1. Description of COMSOL geometry and simulation settings

COMSOL Multiphysics 5.1 was used to simultaneously solve the heat-transfer equations governing the electron and lattice temperatures, as well as the heat transfer to the surroundings. A spherical geometry was designed with the HfN or Au nanodiabolo on an alumina substrate in air, or completely surrounded by H_2O, where the dimensions of the nanodiabolo were variable (length = scale × 194 nm; width = scale × 116.5 nm; height = scale × 40 nm, stereolithographic file (.stl) available for download) and the outer sphere radius (geometry radius = scale × 240 nm), see Figure S5a. A four-fold symmetry was used to minimize computational requirement, with symmetry boundary conditions on the inner boundaries. The geometry was meshed with free tetrahedrons with a maximum element size of scale × 60 nm and a minimum element size of 2 nm, 1.25 maximum element growth rate, 0.3 curvature factor, and 0.85 resolution for narrow regions. To increase surface resolution, the nanoparticle boundaries were meshed with a free triangular mesh at 6× smaller scale than the rest of the geometry. Additionally, to avoid numerical artefacts due to high instantaneous temperature gradients during the fs-laser pulse, 6 boundary layers were applied to the nanoparticle boundaries. At all scales, these settings yielded a tetrahedral mesh consisting of ~95000 individual elements (Figure S5b and c). A time dependent study was employed with two segregated steps using a MUMPS solver: one for solving T_e and T_l, and one for solving the surface chemistry. The error tolerance was set to 1×10^{-7}. To accurately capture all dynamics on fs – ns timescales, while retaining a complete simulation time range of 100 ns, the time stepping was made progressively precise around the pulse time center, set at a delay of t = 3 ns (Table S1), with intermediate time stepping setting. Time-stepping was kept the same, regardless of input conditions (pulse time, energy, material, etc.). The total simulation time was approximately 4 – 5 min on an Intel Core i7-9750H @ 2.60 GHz processor with 16 GB RAM.

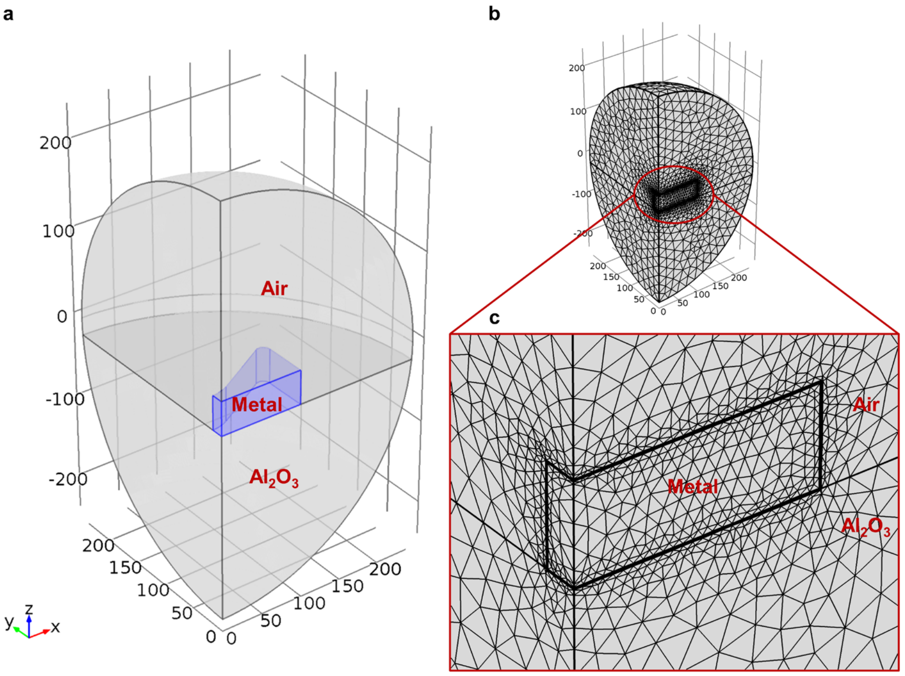

Figure S5. COMSOL simulation geometry (a) and simulation mesh for HfN and Au nanodiabolos (b/c) at 100% particle scale. For simulations in water, the entire outer sphere is set to H_2O. The outer boundary temperature was fixed at 293.15 K throughout the entire simulation.

Table S1. Time stepping settings of the COMSOL simulation.

Time range (ns)	Time step (ps)
0 – 2.5	100
2.51– 2.98	10
2.981 – 2.999	1
2.9992 – 2.9998	0.2
2.99981 – 3.0002 (around laser pulse)	0.01
3.00022 – 3.001	0.02
3.0011 – 3.004	0.1
3.005 – 3.030	1
3.04 – 3.50	10
3.6 – 8.0	100
9000 – 103 000	1000

3.2. Heat transfer and electron-phonon coupling

We use a Dual-Parabolic Two-Step model (DPTS) to fully account for heating and heat transfer contributions in both electron and phonon population.[1] In particular, we choose to explicitly model the heat transfer in the lattice due to the strong coupling of electrons and phonons in this material, which results in strong contribution of both components to the thermal conductivity

at all timescales. Moreover, in HfN the lattice contributes much more to the equilibrium thermal conductivity than in typical metals, which is discussed below. The validity of the DPTS model has been recently demonstrated in the case of transient reflectivity measurements of TiN thin films.[2] Within the HfN nanoparticle domain (the blue domain in Figure S5a) the following coupled partial differential equations apply:

$$C_e(T_e)\frac{\partial T_e}{\partial t} = \nabla[\kappa_e(T_e)\,\nabla T_e] - G(T_e - T_l) + q(x,y,z,t) \qquad \text{Equation 1}$$

$$C_l(T_l)\frac{\partial T_l}{\partial t} = \nabla[\kappa_l(T_l)\,\nabla T_l] + G(T_e - T_l) \qquad \text{Equation 2}$$

where subscripts *e* and *l* distinguish between electronic and lattice terms, *T* is the temperature (in K), *C(T)* is the temperature-dependent volumetric heat capacity (in $J/m^3.K$), κ is the thermal conductivity (in W/m.K), *G* is the electron-phonon coupling constant (in $W/m^3.K$), and q(x,y,z,t) is the pulsed laser heating term (in W/m^3). The initial temperature was set to 293.15 K. The electronic temperature was fully isolated from the air/water surroundings, that is, no electron energy transfer occurs to gas molecules or water. The lattice temperature is fully coupled with the air/water temperature, see below.

In practice, the electron temperature was simulated using the "Heat Transfer in Fluids" module, and the lattice temperature was simulated using the "Heat Transfer in Solids" module. To account for the temperature coupling, both modules were linked using the "Local Thermal Non-Equilibrium" module.[3] This module was originally designed for solid-liquid heat transfer in porous media, in which each phase only occupies a fraction of the volume. Therefore, the underlying equations were rectified to satisfy Equation 1 and Equation 2.

For the air/water and alumina domains, Equation 3 applied with an outside boundary temperature fixed at 293.15 K and an initial temperature set to 293.15 K. Within the model, the air/water/Al_2O_3 temperatures are also defined as "lattice" temperatures (subscript *l*).

$$C_l(T_l)\frac{\partial T_l}{\partial t} = \nabla[\kappa_l(T_l)\,\nabla T_l] \qquad \text{Equation 3}$$

Interfacial heat transfer resistance (Kapitza resistance) at the metal-air/water boundary was not implemented due to the poor availability of reported data. If any significant heat transfer resistance does apply at this interface, the surface will in reality reach higher transient temperatures.[4] Thus, our reported simulation data of the nanoreactor surface temperature and chemical reactivity may be underestimations. Interfacial heat transfer resistance at the metal-Al_2O_3 boundary was not implemented for the same reason. We anticipate that in reality the heat transfer from the nanoparticle to the substrate may be slower due to a finite resistance, making the cooldown slower. The interfacial resistance between TiN and Al_2O_3 has been reported to be very low (conductance ~ 600 MW/m^2.K), and lower in comparison to copper or aluminium and Al_2O_3 (conductance 150-250 MW/m^2.K), so the interfacial resistance may play a bigger role for classic plasmonic metals than for transition metal nitrides.[5,6]

Further, heat transfer through convection was neglected due to the short time-scale of heating and cooling, and due to the small dimensions of the nanoreactor. This was further supported by calculating the Rayleigh number of the diabolo in air and in water by Equation 4, which yields the ratio of heat transfer by convection and diffusion:

$$Ra = \frac{\rho\beta\Delta T_l l^3 g}{\eta\alpha} \qquad \text{Equation 4}$$

with ρ the medium density (water: 1000 kg/m^3; air: 1.2 kg/m^3), β the thermal expansion coefficient (water: 210×10^{-6} K^{-1}; air: 3.4×10^{-3} K^{-1}), ΔT_l the lattice temperature difference (maximum = 220 K directly after excitation for Au at 100% scale in water, 140 K for Au in air), l the fluid size (in this case a 480 nm simulation sphere), g the gravitational acceleration

(9.8 m/s^2), η the dynamic viscosity of the fluid (water: 0.001 kg/m.s; air: 1.8 × 10^{-5} kg/m.s), and α the thermal diffusivity ($\alpha = \kappa_l / \rho C_l$; water: 0.143 × 10^{-6} m^2/s; air: 2.1 × 10^{-5} m^2/s). The Rayleigh number amounts to 2 × 10^{-7} and 2 × 10^{-9} for the reactor in water and in air, respectively. Thus, heat diffusion dominates in both systems, and the slow viscous laminar flow of the environment does not affect the temperature distribution. Heat transfer by convection may become significant with larger assemblies of nanoreactors and with larger illumination areas.

For simulations of nanoreactors in water, and where high peak temperatures were obtained that exceed the spinodal temperature of water (T = 550 K), water vapor bubbles may form around the structure that can influence the heat transfer dynamics.[7] However, such bubble formation is also affected by the Young-Laplace equation, which states that a smaller curvature radius of the bubble results in a higher vapor pressure.[8] Since only a small part of the nanoreactor surface lattice temperature exceeds 550 K, only a tiny bubble is expected, for which the high Laplace pressure would prevent it from forming. The reactor geometry is another factor that can influence bubble formation, and bubble formation temperatures vary greatly for different particles. Finally, the actual temperature of the surrounding water is also greatly affected by the Kapitza resistance (see above).[7] For these reasons it was uncertain whether bubble formation occurs, to what degree, and how it affects heat transfer dynamics and the surface chemistry; therefore it was not implemented at this point.

Finally, heat transfer through radiation was also neglected, because preliminary simulations, for which the Stefan-Boltzmann law of radiation (Equation 5) was applied to the metal nanoparticle boundaries, showed that typically only ~0.001% of the total heat transfer occurred through radiation. For this calculation we assumed a material emissivity of a black body (ε_{emi} = 1).

$$-q_{irr} = \varepsilon_{emi}\sigma(T_{amb}^4 - T_l^4) \quad \text{Equation 5}$$

where σ is the Stefan-Boltzmann constant (σ = 5.67 ×10^{-8} W/m^2.K^4) and T_{amb} is the ambient temperature (293.15 K).

The temperature-dependent electronic heat capacity is calculated using the Debye approximation:

$$C_e(T_e) = \frac{\gamma\rho T_e}{M_W} \quad \text{Equation 6}$$

where γ is the electronic heat capacity (also known as the Sommerfeld constant; for HfN γ = 1.2 × 10^{-3} J/mol.K^2),[9] ρ is the density (13680 kg/m^3), and M_W is the molar weight (M_w = 0.1925 kg/mol). This approximation holds well for noble metals in the low electron temperature regime ($0 < T_e < 0.1T_F$, with T_F being the Fermi temperature, that is $T_F = E_F/k_B \approx 6 \times 10^4$ K)[10], and holds for group 4b transition metal nitrides as well.[11]

The temperature-dependent heat capacity of HfN has been reported from 5.6 – 346 K,[9] which is in agreement with theoretical works[12,13] and the tabulated value at 293 K (38 J/mol.K, that is 2.70 × 10^5 J/m^3.K).[14] These tabulated data were extracted, fitted with the 6th-order polynomial in Equation 7, and used for $C_l(T_l)$. We assumed a negligible electronic component in this data, due to the much lower heat capacity of electrons compared to phonons. Indeed, this is justified by realizing that the magnitude of $C_e(T_e)$ at any temperature is less than 2% of $C_l(T_l)$, see Figure S6a and b.

$$C_l(T_l) = -5.33 \times 10^5 + 2.41 \times 10^4 T_l - 67.5T_l^2 + 0.109T_l^3 - 9.85 \times 10^{-5}T_l^4 + 4.62 \times 10^{-8}T_l^5 - 8.72 \times 10^{-12}T_l^6 \quad \text{Equation 7}$$

For metals, the overall thermal conductivity is given by the linear combination of electronic and lattice contributions, with weights a and b, respectively:

$$\kappa(T) = a\kappa_e(T_e) + b\kappa_l(T_l) \quad \text{Equation 8}$$

The overall thermal conductivity of HfN was extracted from literature,[15,16] which is in good agreement with the Handbook of of Refractory Carbides and Nitrides (κ(293 K) = 21.7 W/m.K)[14], and fitted with the polynomial in Equation 9, plotted in Figure S6c. The thermal conductivity of HfN is considerably lower than that of Au (κ(293 K) = 318 W/m.K). We also note that the extracted thermal conductivity of HfN includes the effect of material defects, which lower the thermal conductivity substantially. It was shown that the theoretical value of defect-free HfN is approximately 90 W/m.K at 300 K.[17]

$$\kappa(T) = 14.02 + 0.01697T - 5.33 \times 10^{-6}T^2 \quad \text{Equation 9}$$

For metals such as gold, the overall thermal conductivity stems primarily from the electrons, so that $a \approx 0.99$, while the lattice contributes very little, so that $b \approx 0.01$.[1] However, in the case of HfN, it was recently shown that the phonon contribution is much larger, in the order of 25%.[17] Therefore, for transition metal nitrides we used $a = 0.25$ and $b = 0.75$:

$$\kappa_e(T_e) = 0.25\kappa(T) \quad \text{Equation 10}$$

$$\kappa_l(T_l) = 0.75\kappa(T) \quad \text{Equation 11}$$

For simulations on Au, the built-in lattice heat capacity and thermal conductivity from COMSOL was used (plotted in Figure S6), while the electronic heat capacity was calculated using Equation 6, where $\frac{\gamma\rho}{M_W}$ = 71.4 J/m^3.K (= 0.729 × 10^{-3} J/mol.K^2).[18]

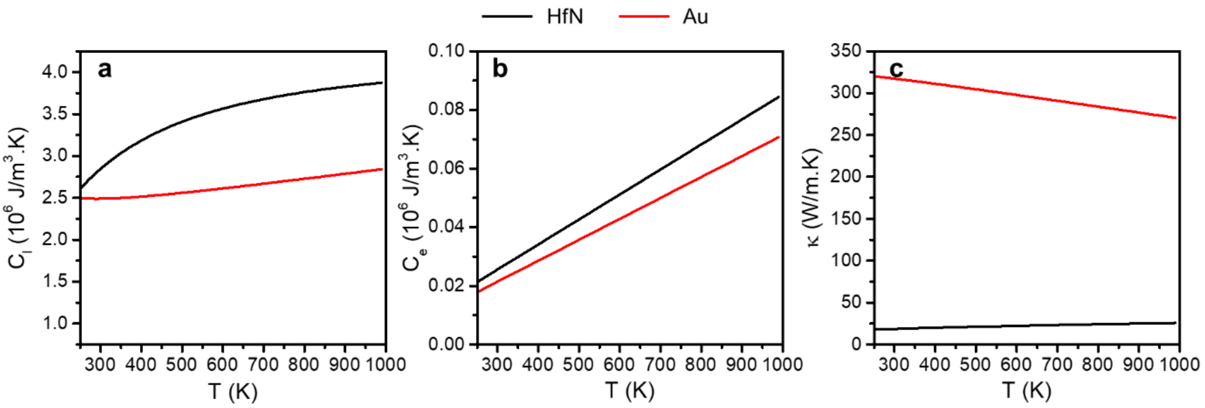

Figure S6. Lattice heat capacity (a), electronic heat capacity (b), and total thermal conductivity (c) of HfN (red curves) and Au (black curves).

The electron-phonon coupling constant *G* was calculated from literature values by using the following approximation, which has been derived by Allen in 1987,[19] and later used for TiN thin film experiments[2] and in theoretical work.[11] We here derive *G* for HfN by using Equation 12:

$$G = \frac{3\gamma\lambda\langle\omega_2\rangle\rho}{\hbar\pi k_B M_W} \qquad \text{Equation 12}$$

where γ is the electronic heat capacity (also known as the Sommerfeld constant; for HfN γ = 1.2 × 10^{-3} J/mol.K^2), λ is the Bardeen-Cooper-Schrieffer electron-phonon coupling constant (0.643 for HfN, no units),[20] $\langle\omega_2\rangle$ is the second moment of the phonon spectrum (in J^2), ρ is the material density (13680 kg/m^3), $\hbar$ is the reduced Planck's constant (6.626 × 10^{-34}/2π J.s), k_B is the Boltzmann constant (1.38 × 10^{-23} J/K) and M_W is the molar weight of HfN (0.1925 kg/mol). The combined terms $\lambda\langle\omega_2\rangle$ is also known as the Eliashberg function. Although a value for $\langle\omega_2\rangle$ is not readily available for HfN in literature, it is possible to estimate $\langle\omega_2\rangle$ from the Debye temperature θ_D^0 (421 - 484 K,[9,14,20] 491 K theoretical prediction[21]) according to Equation 13:[2,22]

$$\langle\omega_2\rangle = (\theta_D^0 k_B)^2 = 3.4 \times 10^{-41} - 4.5 \times 10^{-41} J^2 \qquad \text{Equation 13}$$

Thus, Equation 12 becomes Equation 14:

$$G = \frac{3\gamma\lambda(\theta_D^0)^2 k_B \rho}{\hbar\pi M_w} = 1.2 \times 10^{18} - 1.6 \times 10^{18}\ W/m^3K \qquad \text{Equation 14}$$

Such high G values in the order of 10^{18} are two to three orders of magnitude higher than measured values reported for Au: 1.1 – 4 × 10^{16} W/m^3.K.[23] For simulation purposes we use an averaged value of 1.4 × 10^{18} W/m^3.K. Here, Equation 14 was also used to calculate G for Au, which resulted in a value that falls accurately within the reported range (2.78 × 10^{16} W/m^3.K, see Table S2). The high G values for HfN are in line with what has been recently reported for TiN and ZrN in literature.[2,11,24] A high G-value immediately indicates short hot carrier lifetimes. Although the value of G varies with electronic temperature,[11] within the temperature range in this work it can be approximated as a constant value. Simulation parameters used for other metals (Ag, Cu, Al, TiN, Rh, Pd, Pt, and Ru) are listed in section 5, Table S3.

Table S2. Summary of parameters used to calculate the electron-phonon coupling constant (G) for HfN and Au.

**Material**	**HfN**	**Au**
γ (J/mol.K^2)	1.2 × 10^{-3} [9]	0.729 × 10^{-3} [18]
a	0.25 [17]	0.01 [1]
b	0.75 [17]	0.99 [1]
λ	0.643 [20]	0.13 [25]
$\langle\omega_2\rangle$ (J^2)	3.4 × 10^{-41} – 4.5 × 10^{-41} (calc.)	4.57 × 10^{-42} [25]
θ_D^0 (K)	421 – 484 [9,14,20]	170 [18]
ρ (kg/m^3)	13680	19320
M_W (kg/mol)	0.1925	0.1970
G (10^{17} W/m^3.K)	14 (calc.)	0.278 (calc.)

3.3. Optical heat source

The spatial and temporal heating profile q(x,y,z,t) originating from laser pulse excitation was modelled as follows. First, finite difference time domain (FDTD) simulations were conducted using Lumerical software to solve Maxwell's equations for the nanoparticle geometry, under broadband plane-wave excitation (λ = 300 – 3000 nm). Optical constants for HfN were used from previous work in which high quality thin film HfN was studied (Figure 1, main text).[26]

For Au, the optical constants from Johnson and Christy were used.[27] The background refractive index was set to 1.000 (air) or 1.333 (H_2O). Four-fold axial symmetry was used. A cross section monitor was used to obtain the absorption and scatter cross sections (σ_{abs} and σ_{scat}), and an electric field monitor was used to visualize the field enhancement around the nanoparticle at peak resonance wavelength (see Figure S1 to Figure S4). An advanced absorbed power monitor was used to retrieve the three-dimensional absorbed power profile within the nanoparticle ($\bar{P}_{abs}(x,y,z)$), which is proportional to the product of electric field intensity (E^2) and the imaginary part of the permittivity (ε''):

$$\bar{P}_{abs}(x,y,z) = -0.5\omega|E(x,y,z)|^2\varepsilon'' \quad \text{Equation 15}$$

where E(x,y,z) is the spatial electric field distribution and ω is the light frequency. To normalize the absorption profile, all output values were divided by the global average within the nanoparticle domain to yield $\bar{P}_{abs}(\mathrm{x,y,z})$, which was subsequently imported in Comsol as an interpolated function, with linear interpolation and an extrapolated value of 0. A plot of $\bar{P}_{abs}(\mathrm{x,y,z})$ is given in Figure S1 and Figure S3 for air and water surroundings, respectively. The peak heating power for the single nanoreactor (q_{peak} in W) was set at a realistic power for low-intensity unfocussed fs-laser irradiation conditions, unless otherwise specified: average laser beam power (P_{ave} = 0.25 mW), laser repetition frequency (f_{rep} = 2.5 kHz), laser pulse full width half maximum (FWHM = 50 fs), laser beam area (beam radius = 100 µm, i.e. A = 0.0314 × 10^{-6} m^2), and absorption cross section (σ_{abs}):

$$q_{peak} = 0.94\frac{P_{ave}\sigma_{abs}}{f_{rep} \times A \times FWHM} \quad \text{Equation 16}$$

Finally, q(x,y,z,t) was obtained by multiplying the product of q_{peak} and $\bar{P}_{abs}(\mathrm{x,y,z})$ by a normalized gaussian temporal pulse (with σ = FWHM/2.3548), which was centered at 3 ns for practical purposes. To account for the four-fold geometrical symmetry, q_{peak} was divided by 4.

$$q(x,y,z,t) = e^{\frac{(t-3\times10^{-9})^2}{2\sigma^2}} \times \frac{q_{peak}}{4} \times \bar{P}_{abs}(x,y,z) \qquad \text{Equation 17}$$

For continuous wave simulations, a constant heat source was applied, with the heating profile for a single nanoreactor given by Equation 18 and Equation 19:

$$q(x,y,z,t) = \frac{q_{cw}}{4} \times \bar{P}_{abs}(x,y,z) \qquad \text{Equation 18}$$

$$q_{cw} = \frac{P_{ave}\sigma_{abs}}{A} \qquad \text{Equation 19}$$

3.4. Thermally activated chemical reaction

On the surfaces that are in contact with the air surroundings, a first-order thermally activated reaction was taking place (Reactant → Product). The kinetics of this reaction were governed by Equation 20 and Equation 21:

$$\frac{d[Product]}{dt} = k[Reactant] \qquad \text{Equation 20}$$

$$k = A \times \frac{T_l}{T_0} \times e^{\frac{-E_a}{RT_l}} \qquad \text{Equation 21}$$

Where A is the pre-exponential Arrhenius factor, which was set to 1×10^{14} s^{-1} as a realistic value for typical unimolecular surface reactions,[28] T_0 is the temperature before light absorption (293.15 K), E_a is the activation energy, which was arbitrarily set to 1 eV (96 kJ/mol) and was kept constant for each material (unless otherwise specified), and R is the gas constant (8.3145 J/K.mol). The initial reactant concentration was set to 1.66×10^{-5} mol/m^2 (10^{19} atoms per m^2, a typical value for metal surfaces), which can be regarded as a fully saturated surface with a monolayer of reactant. No migration or diffusion was incorporated in the model, and no exchange of molecules took place between gas or liquid phase and adsorbed phase. Further, the reaction occurs without change in enthalpy, that is, no heat is either consumed or generated.

Because the metallic nanoreactor surface heats up to ~1000 K, the pre-exponential factor is no longer independent of temperature. Therefore, the Arrhenius exponential term was modified using a moderate temperature dependence (T_l / T_0).[29] Under these conditions, reactant depletion did not occur in our experiments: typically, for HfN at 40% scale, approximately 0.5% of the reactant was converted at the optical hotspot (for 0.32 mJ/cm^2 irradiation (0.8 W/cm^2), 50 fs pulse FWHM). In all data we subtract the rate of production in the dark, *i.e.* at 293.15 K, to obtain a strictly photo-induced conversion yield. For comparison, the amount of dark product for the nanoreactors at 40% scale is 2.6×10^{-5} molecule per 100 ns, or 0.10 molecule per 400 μs (which is the pulse to pulse time at 2.5 kHz excitation). The total product yield was evaluated after 100 ns simulation time (unless otherwise specified) by integrating the product density (molecules/nm^2) over the total surface area of the nanoreactor that was in contact with the air or water surroundings. The product formation selectivity at the optical hotspot was evaluated by taking the ratio of the averaged product density at the waist line of the diabolo and the averaged product density at the outer edge of the diabolo (parallel to the waist line).

We do not consider the participation of plasmonic hot carriers on chemical activation. For transition metal nitrides the hot carrier lifetime is very short, which may greatly reduce the chances of participation in chemical reactions. This also suggests that only ballistic hot carriers would be expected to participate, directly after photoexcitation and before they scatter. However, ballistic hot carriers in transition metal nitrides have also been calculated to have much shorter lifetime than in Ag ($\tau < 10$ fs and decreasing greatly with higher carrier energy).[30] Thus, hot carrier effects are less likely to occur than for noble metals. For noble metals and other plasmonic metals (Pd, Rh, Ru, Pt, etc) these effects may be greater, providing alternative and complementary routes for chemical activation.[31]

It is important to note that we here assume that the chemical reaction occurs instantaneously, while in reality the molecule undergoes a transition from reactant to product through a transition

state, each of which steps taking a certain time to complete. First, upon lattice heating of the nanoreactor, multiple phonons need to be transferred to the molecular reactant in order for it to reach a higher molecular vibrational state; this may take several ps.[32–34] Thus, a vibrational energy exchange exists between nanoreactor and molecules, which produces a dynamic distribution in vibrationally excited molecules. As the surface cools down, this distribution will also change accordingly. Vibrational excitation of adsorbates is likely to occur faster for transition metal nitride nanoreactors due to the faster electron-phonon coupling time, because phonons are available practically instantaneously after light excitation. Secondly, although chemical bonds vibrate at 15 – 90 THz frequencies (500 – 3000 cm^{-1}), *i.e.* with 11 – 67 fs oscillations, elementary chemical reactions occur on a somewhat longer timescale (typically several ps),[34,35] in which timespan the molecule makes many attempts to traverse the potential energy landscape and cross the energy barrier from reactant to product. An important implication is that if the transient thermal hotspot would exist for only a very short time, there may not be enough time to complete the reaction: vibrational energy will be lost to the environment before any reaction can occur. However, in the main text we demonstrate that the typical duration of transient thermal hotspots are several hundreds of ps, much longer than the timescale of the elementary chemical step. Concluding, inaccuracies in our model are most likely to occur in the fs to 10 ps timespan, where our assumptions fall short. This may be in the future accounted for by using our 3D two-temperature model in combination with, for instance, DFT approaches or multi-scale Monte Carlo modelling for specific chemical reactions,[36] supported with experimental time-dependent optically-pumped x-ray techniques.[34]

4. Two-temperature heat transfer modelling

4.1. Au nanorod on Al_2O_3 in air

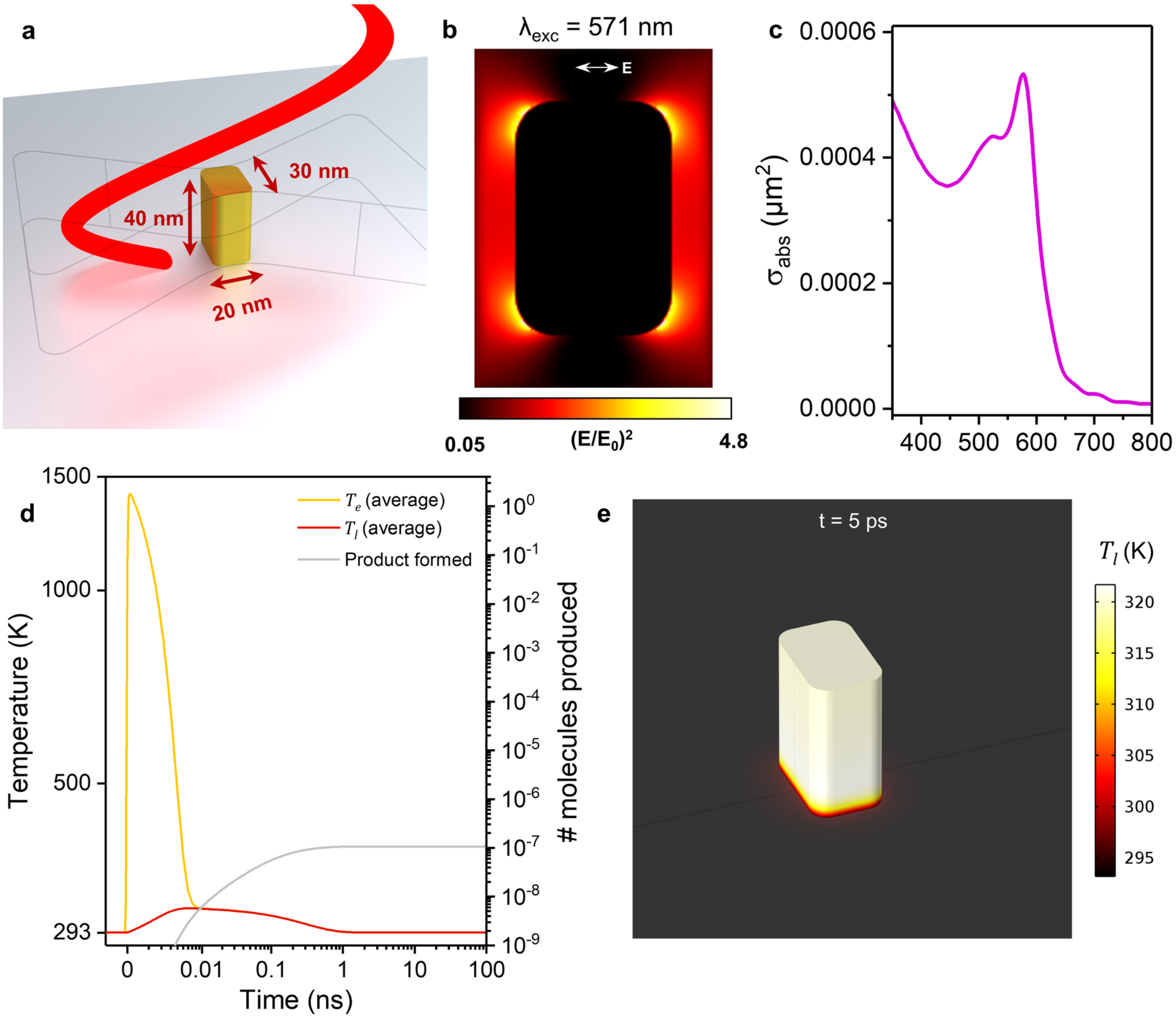

Figure S7. Optical and heat transfer simulations of a Au nanorod with the size of the nanodiabolo constriction (at 100% scale). a) Dimensions of the Au nanorod. The nanodiabolo at 100% scale is shown as line contours. b) FDTD-simulated electric field map (at z = 20 nm). The simulation was performed with plane wave excitation polarized along the short axis of the nanorod (x-axis) and at the peak absorption wavelength (571 nm). c) Absorption cross section spectrum. d) Time-evolution of volume-averaged electronic (yellow) and lattice temperatures (red), and the number of product molecules produced per pulse (grey) during 50 fs pulsed excitation at 0.32 mJ/cm^2 pulse energy (0.8 W/cm^2) for the Au nanorod reactor (λ_{exc} = 571 nm). e) Lattice temperature at t = 5 ps.

4.2. HfN and Au diabolo on Al_2O_3 in air

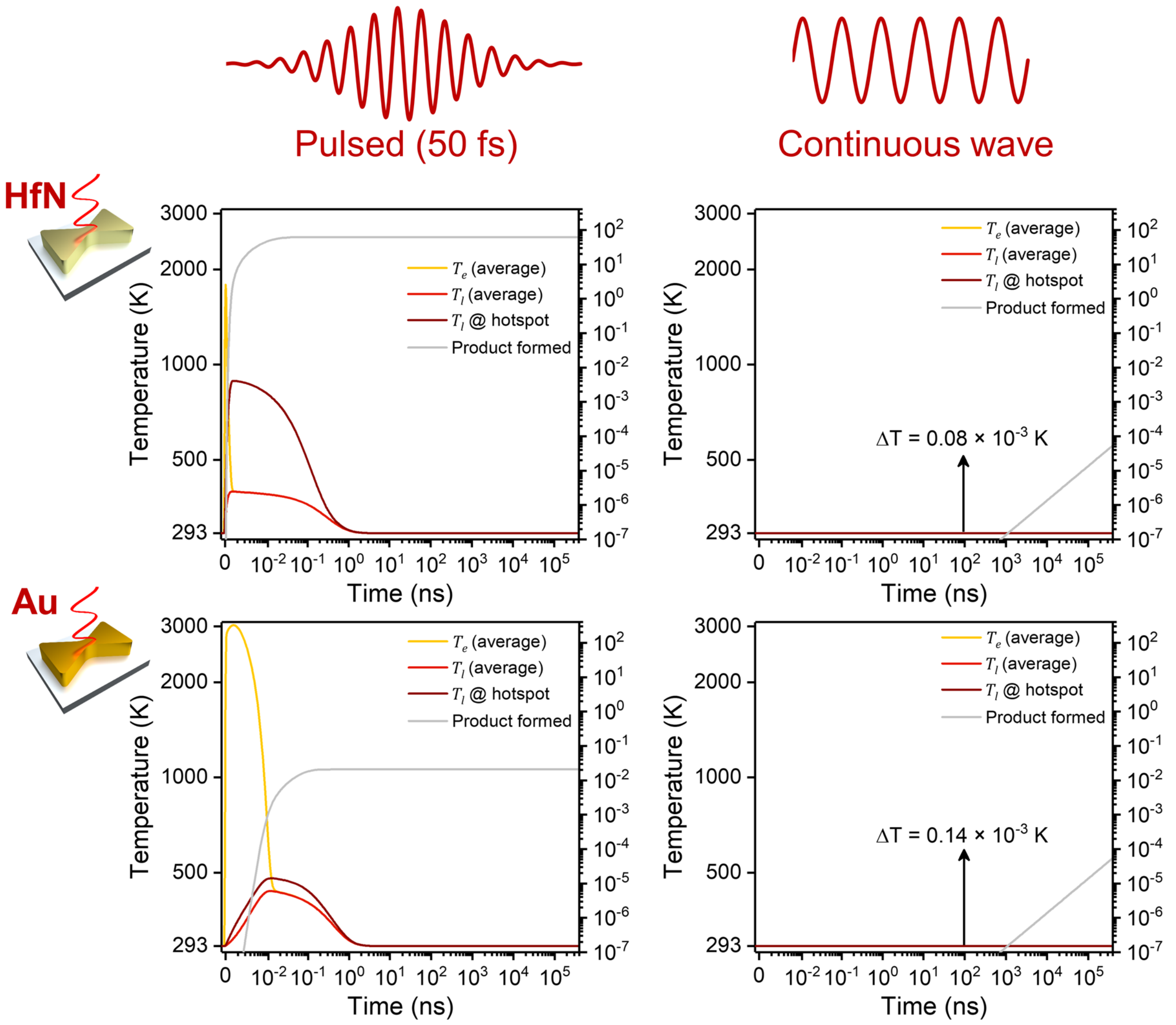

Figure S8. Comparison of pulsed (left) and continuous wave (right) excitation for HfN (top) and Au (bottom) nanoreactors at 100% scale, with an identical average power of 0.8 W/cm^2 (0.32 mJ/cm^2 pulse energy at 2.5 kHz). The full timespan from pulse to pulse was covered (400 µs). As mentioned in section 3.4, the dark rate was subtracted from the data, which amounts to 0.10 molecules per 400 µs. Note that the product yield for Au (CW & pulsed) and CW HfN are much lower than this dark production rate. In contrast, the pulsed-excited HfN nanoreactor produces 62 molecules per pulse, or 620 times the amount produced in absence of light.

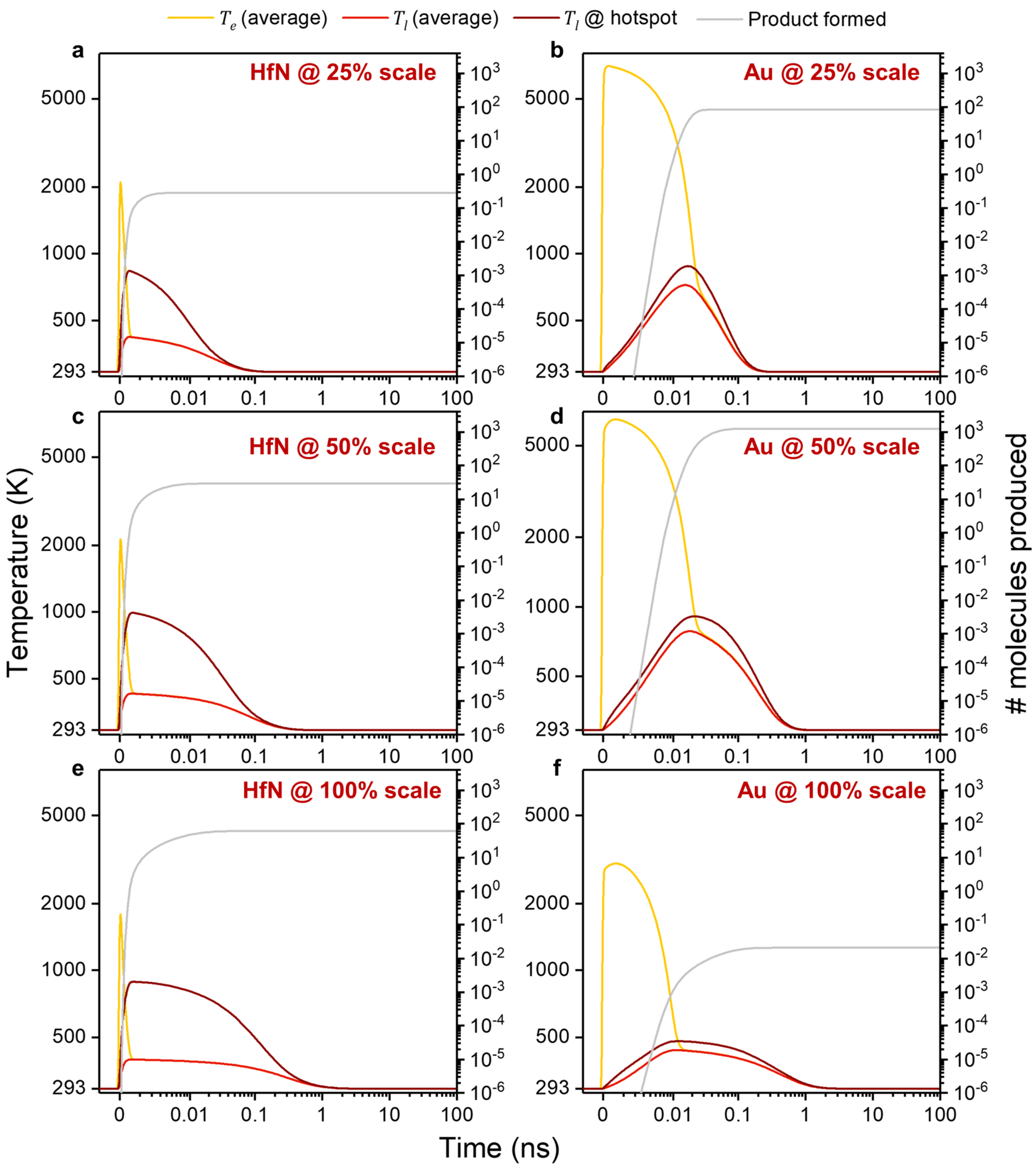

Figure S9 (continues on next page).

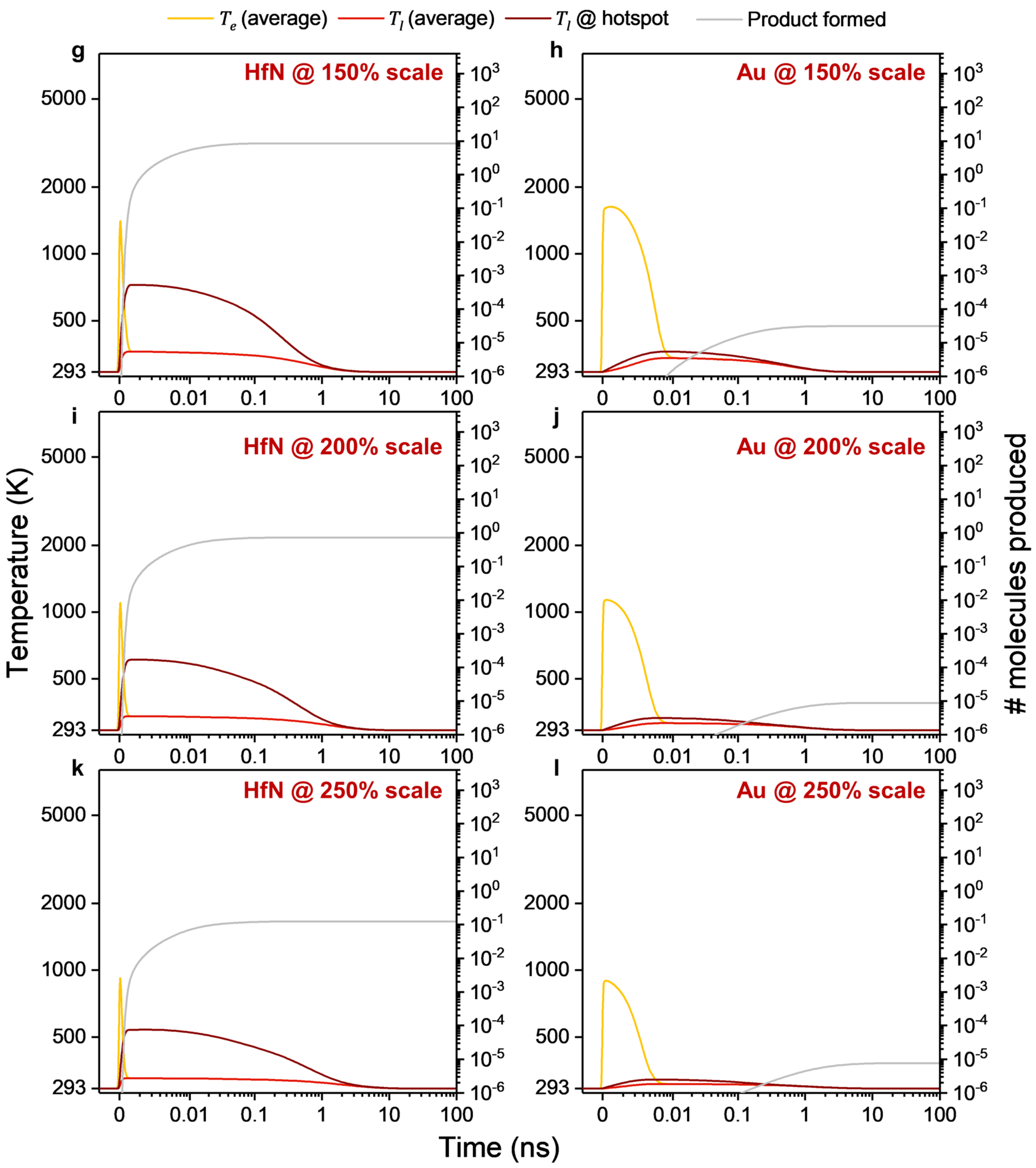

Figure S9 continued. Time-evolution of volume-averaged electronic (yellow) and lattice temperatures (red), the lattice temperature at the absorption hotspot (dark red), and the number of product molecules produced per pulse (grey) during 50 fs pulsed excitation at 0.32 mJ/cm^2 pulse energy (0.8 W/cm^2) for HfN (left panels a, c, e, g, i, and k) and Au (right panels, b, d, f, h, j, and l) nanoreactors on Al_2O_3 substrate and in air, at 25 – 250% scale, as indicated in each panel.

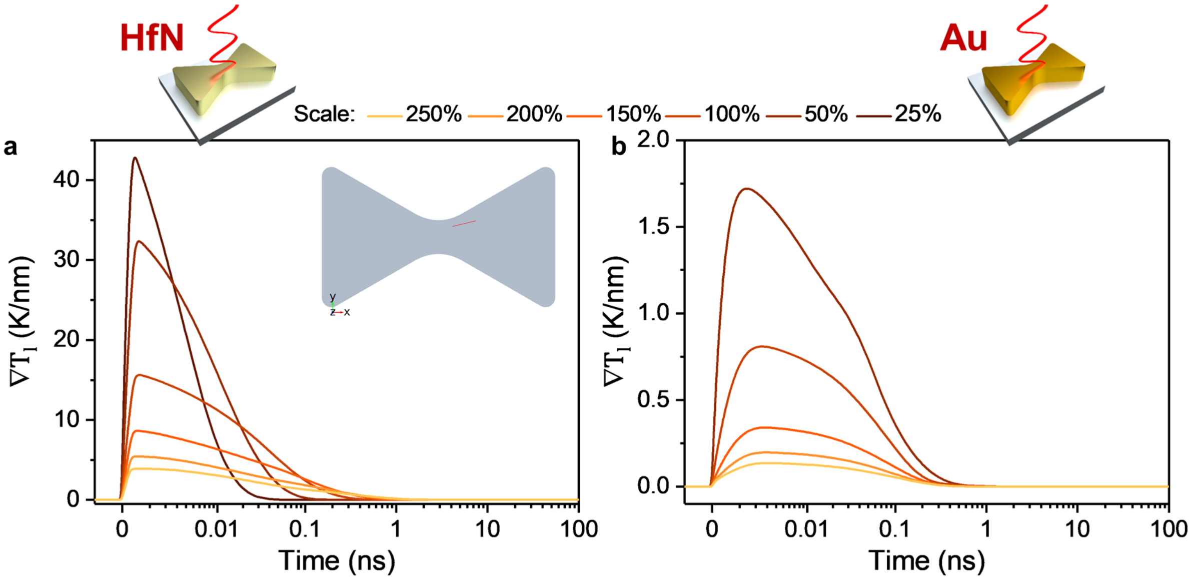

Figure S10. Temporal decay of surface thermal gradients of HfN (a) and Au diabolos (b) at 25 – 250% scale (dark red to yellow) after excitation with 50 fs pulsed irradiation at 0.32 mJ/cm^2 pulse energy density (0.8 W/cm^2). The gradient was measured as the average value along the red line in the inset, where the gradient is typically maximal (Figure S10). Note the different y-axis scale for panels a and b. For the Au nanoreactor at 25% scale, no gradient was present (*i.e.* there was no transient thermal hotspot at all), and was thus omitted from this overview.

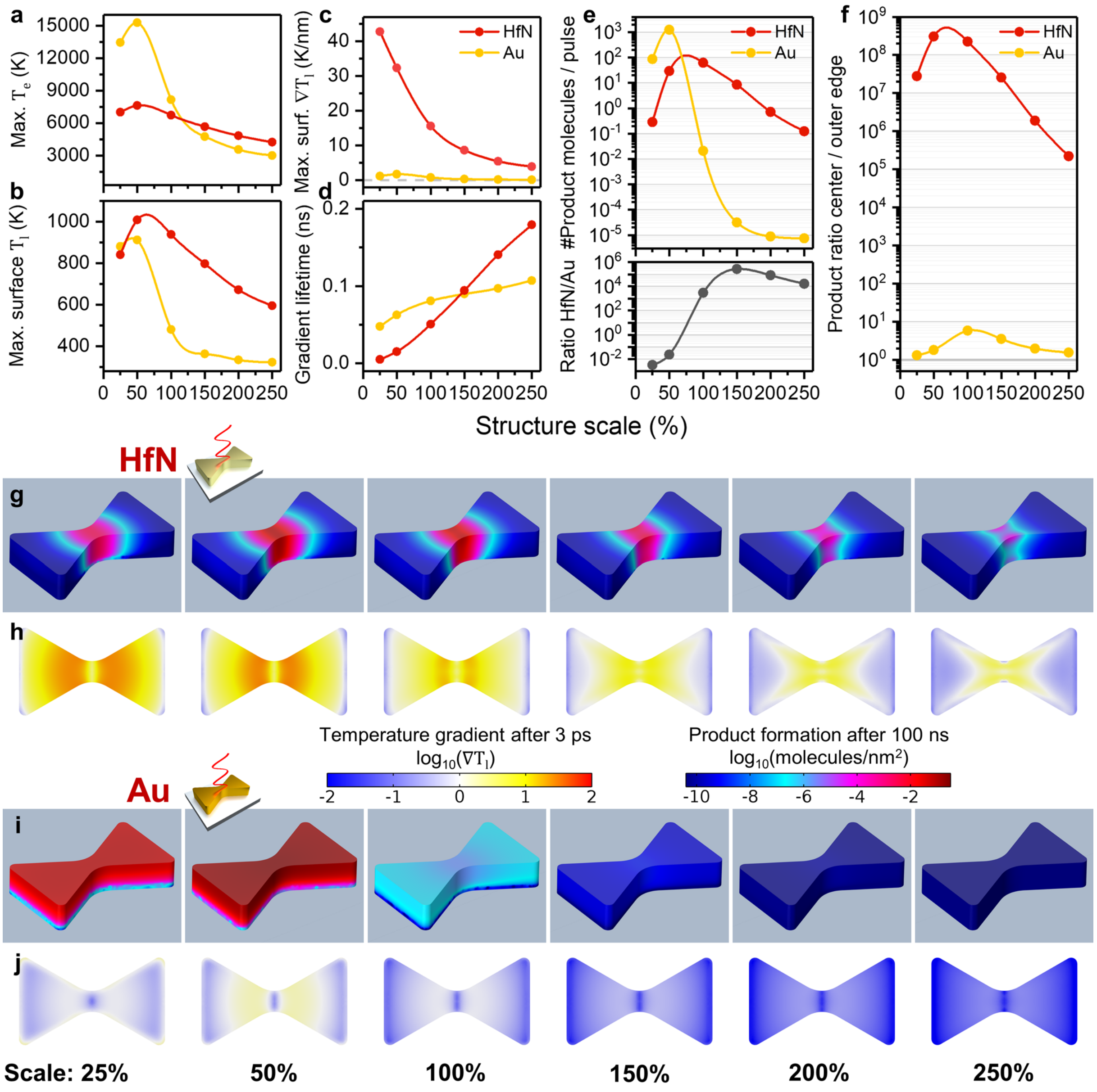

Figure S11. Influence of structure scale on maximum electronic temperature (a), maximum surface lattice temperature (b), maximum top-surface lattice temperature gradient (c, also see Figure S9), mean lifetime of this gradient (d), amount of product molecules per pulse (e), and spatial selectivity of the product formation (f) for HfN (red) and Au (yellow) nanoreactors. g&i) Chemical product formation maps after 100 ns simulation time in logarithmic color scale for HfN (g) and Au (i) nanoreactors at 25 – 250% scale. h&j) Top-surface temperature gradient after 3 ps simulation time in logarithmic color scale for HfN (h) and Au (j) nanoreactors at 25 – 250% scale. The temperature gradient images have two reflection symmetry axes: one passing through the center of the diabolo horizontally and the other vertically. The reader is encouraged to view Supporting Video 1 to compare temperature dynamics and product formation at each scale.

4.3. HfN and Au diabolo in H_2O

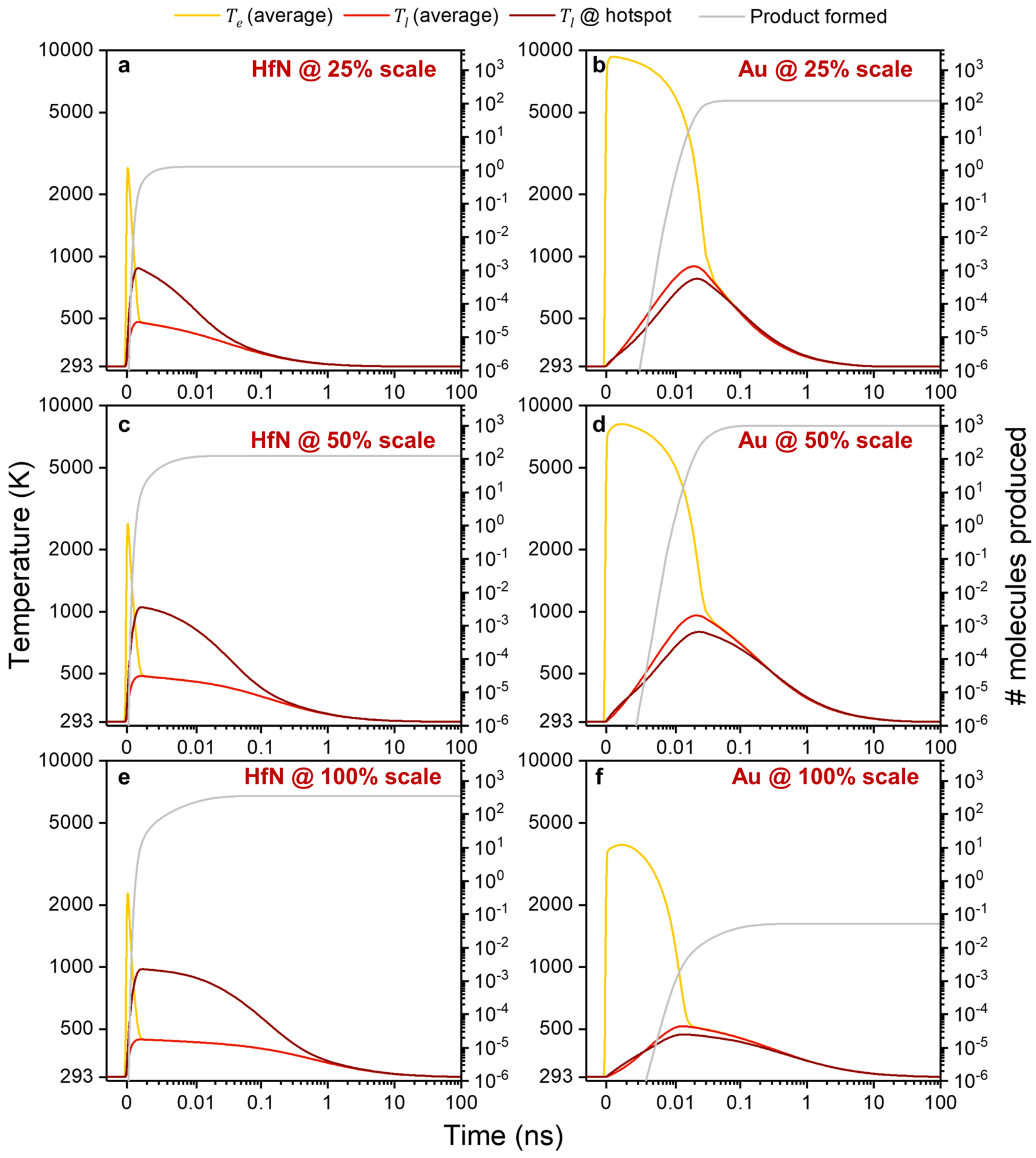

Figure S12 (continues on next page).

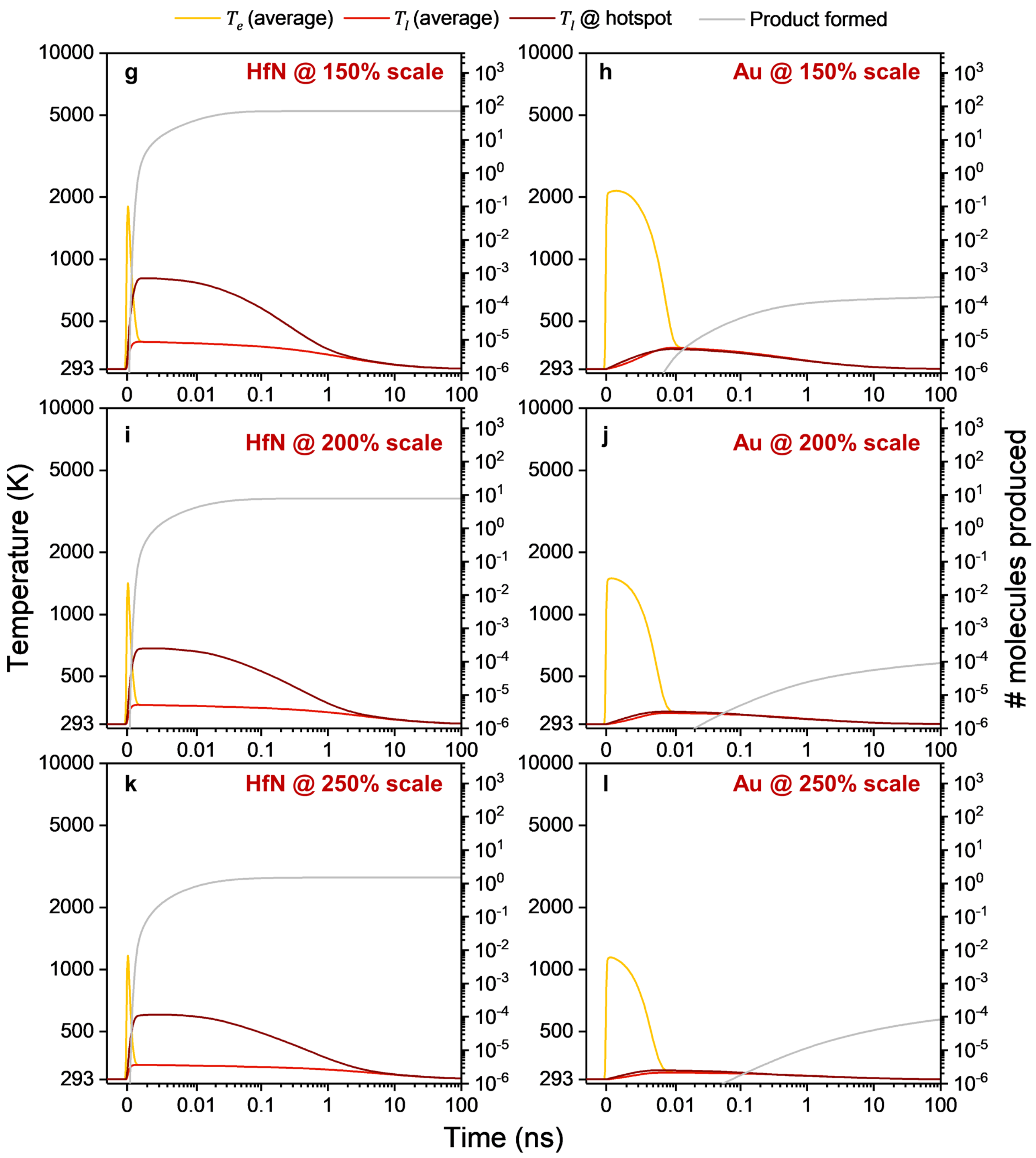

Figure S12 continued. Time-evolution of volume-averaged electronic (yellow) and lattice temperatures (red), the lattice temperature at the absorption hotspot (dark red), and the number of product molecules produced per pulse (grey) during 50 fs pulsed excitation at 0.32 mJ/cm^2 pulse energy (0.8 W/cm^2) for HfN (left panels a, c, e, g, i, and k) and Au (right panels, b, d, f, h, j, and l) nanoreactors in H_2O surroundings, at 25 – 250% scale, as indicated in each panel. Note the longer timespan needed to completely cool down back to 293.15 K, compared to simulations on Al_2O_3 substrate (Figure S9). Also, in absence of a thermally conductive substrate, the lifetime of the transient thermal hotspot is extended, reaching several ns for the largest nanoreactor sizes. These two factors boost the product yield for both materials approximately ten-fold.

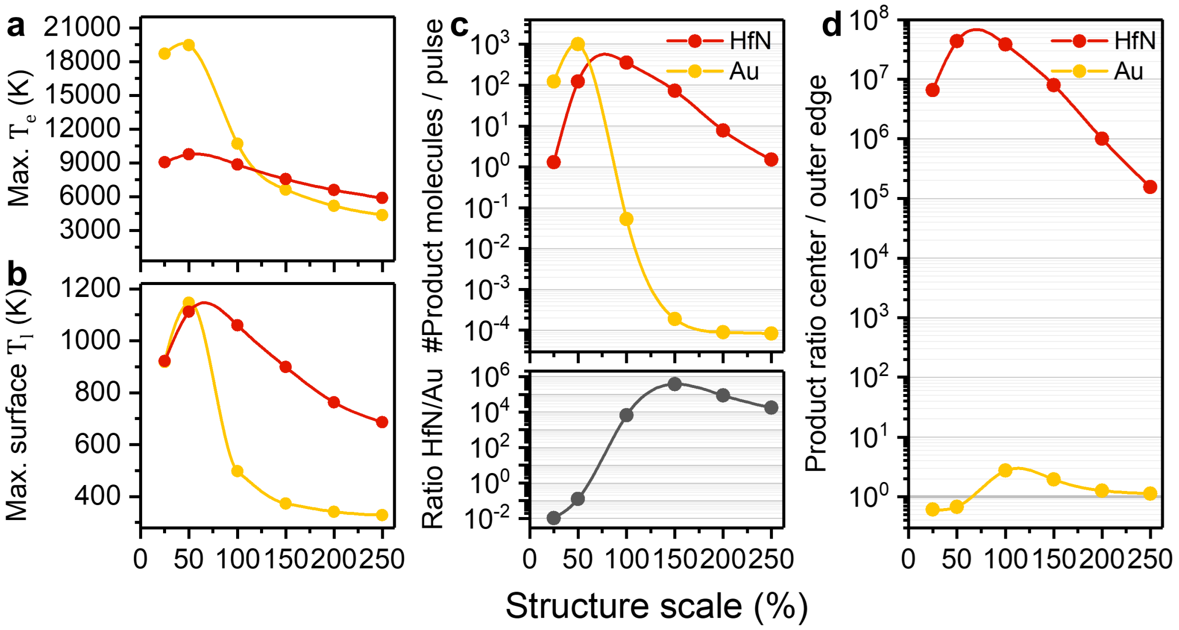

Figure S13. Influence of structure scale on maximum electronic temperature (a), maximum surface lattice temperature (b), amount of product molecules per pulse (c), and spatial selectivity of the product formation (d) for HfN (red) and Au (yellow) nanoreactors in H_2O surroundings. The results mirror those obtained for nanoreactors on Al_2O_3 and in air, although there are quantitative differences. Because of the absence of a thermally conductive substrate, the transient thermal hotspot lives longer and the total nanoreactor cools down slower to room temperature (see Figure S12), which in turn boosts the product yield ten-fold. The reader is encouraged to view Supporting Video 2 to compare temperature dynamics and product formation at each scale.

4.4. Discussion on the effect of nanoreactor scale

The time-dependent temperature dynamics and chemical product formation of HfN and Au nanoreactors at 25 – 250% scale are shown in Figure S9 and Supporting Video 1, where the absorption cross section and excitation wavelength were adjusted for material and size, according to Figure S2. These data are summarized in Figure S11, which shows, as a function of nanoreactor scale, the maximum volumetric electronic temperature, maximum surface lattice temperature, product formation after 100 ns simulation time, and the product formation selectivity at the absorption hotspot.

For small structures at 25% and 50% scale (48.5 and 97 nm length, respectively), Au absorbs 4.8 and 3.5× more than HfN, respectively, which causes the electronic temperature to rise to >7000 K, compared to 2000 K for HfN. As a result of the high absorption per unit volume, electron-phonon equilibration in Au takes more than 25 ps to complete.[37] Within this timeframe, due to the high electronic thermal conductivity (99 % of the overall thermal conductivity), the electronic heat is spread isotropically throughout the entire nanoreactor. Consequentially, the lattice heats up isotropically to 722 (max. 880 K) and 786 K (max. 912 K) at 25% and 50% scale, respectively, approaching the melting point of an Au nanoparticle surface.[8] Such temperatures are likely to be unsustainable for intricate Au nanostructures, which are expected to deform. The heat-transfer dynamics for HfN nanoreactors are not very different at 25 – 50% scale compared to 100% scale, with a clear difference between lattice temperature at the hotspot and at the outer plane of at least 400 K (max. 841 K at 25% scale and 1008 K at 50% scale). Thus, the HfN nanoreactor reaches comparable temperatures at the hotspot as that the Au nanoreactor reaches at the entire surface. For both materials, the internal lattice temperature equilibration (equal temperature at hotspot and outer plane) takes much shorter times to complete than at 100% scale. In terms of product formation the Au nanoreactors produces 300 and 42× more chemical product at 25% and 50% scale, respectively. However, the product

formation in Au nanoreactors is almost completely isotropic (very low selectivity for the optical absorption hotspot of ≤ 1.8), while highly specific at the hotspot for HfN (≥ 3 × 10^7), see Figure S11f/g/i.

At 150%, 200%, and 250% scale, the tables are turned: HfN absorbs 1.8×, 2.5×, and 2.9× more light, respectively, which leads to a >1000 K higher maximum T_e than Au at each scale. Transient thermal hotspots are formed for HfN, but only very weakly for Au, which expresses itself as a 272 – 434 K higher maximum surface T_l observed for HfN nanoreactors: 798 K, 673 K, and 596 K for HfN, and 364 K, 335 K, and 324 K for Au, at 150%, 200%, and 250% scale, respectively. Compared to the nanoreactor at 100% scale, cooldown to room temperature takes up to 10 ns to complete. As a consequence of the transient thermal hotspot, the HfN nanoreactors all yield >10^4 more product than the Au nanoreactor. Just like for smaller nano-reactors, the selectivity of the chemical reaction for the absorption hotspot was very high for HfN nanoreactors (>10^5), but very weak for Au nanoreactors (≤ 3.5), see Figure S11f/g/i.

The same qualitative trends were observed when the nanoreactors were free-floating in water (Figure S12, Figure S13, and Supporting Video 2). Quantitative differences originated from a trade-off between ~50% higher absorption cross sections in H_2O surroundings (Figure S4), and the strong surface cooling effect of water (high heat capacity), which in turn dampens the chemical surface reaction. The net result is a ten-fold increase in chemical yield for both materials. Furthermore, in absence of a thermally-conductive Al_2O_3 substrate the reactors cool down much slower. The use of thermally isolating substrates (e.g. glass) will thus retain heat much better at the nanoreactor. However, since the nanoreactor is isotropically heated in the last phase of cooldown, thereby driving surface chemistry equally isotropically, a longer cooldown period will likely lower the reaction selectivity for the optical hotspot. Overall, these results showed that the transient thermal hotspot strategy is a general phenomenon and is applicable to both substrate-adhered nanoreactors or free-floating nanoreactors, regardless of

surrounding medium. Tuning of the medium and substrate are important to achieve maximum product yield or maximum localization.

Additionally, the temperature gradient on the top surface was analyzed and visualized, shown in Figure S10 and Figure S11c/d/h/j. At all scales, enormous transient temperature gradients are observed for HfN nanoreactors, with values increasing from 3.9 K/nm at 250% scale to 43 K/nm at 25% scale, systematically higher than those in Au (0.1 K/nm at 250% scale and 1.2 K/nm at 25% scale). The lifetime of the gradients varied strongly with size, but reached 100 – 200 ps in HfN at 150 – 250 % scale. Thus, even though the thermal gradients from hotspot to outer edge are much higher for HfN, the lifetimes are comparable due to the large difference in thermal conductivity. These high transient thermal gradients can be exploited in nanoscale photo-thermoelectric devices, could prove useful in heat assisted magnetic recording and would have completely unknown effects in chemical reactions. For instance, the fast heating and cooling down of adsorbates at transient thermal hotspots enables the initiation of catalytic cycles and the analysis of kinetically trapped intermediates using pump-probe spectroscopy.

Taken altogether, the data show that size and absorption cross section of the nanoreactor have a profound influence on the performance of the nanoreactor, with large observed differences in product formation, magnitude of thermal gradient, and lifetime of thermal gradient. Transient thermal hotspots do not always outcompete noble metals in terms of product yield: for diabolo nanoreactors our results predict that transition metal nitrides outcompete noble metals when at least half of the absorption is achieved (at approximately >75% scale, see Figure S11e). Thus, material, nanoreactor geometry, and device configuration need to be carefully optimized for maximization of absorption. However, when high thermal gradients or hotspot specificity is required, transition metal nitrides are always the best choice, regardless of scale. Concluding, the results demonstrate that intense transient thermal hotspots with high associated thermal gradients can be robustly generated by pulse-exciting any size HfN nanoreactor.

5. FDTD and heat transfer modelling for other metals

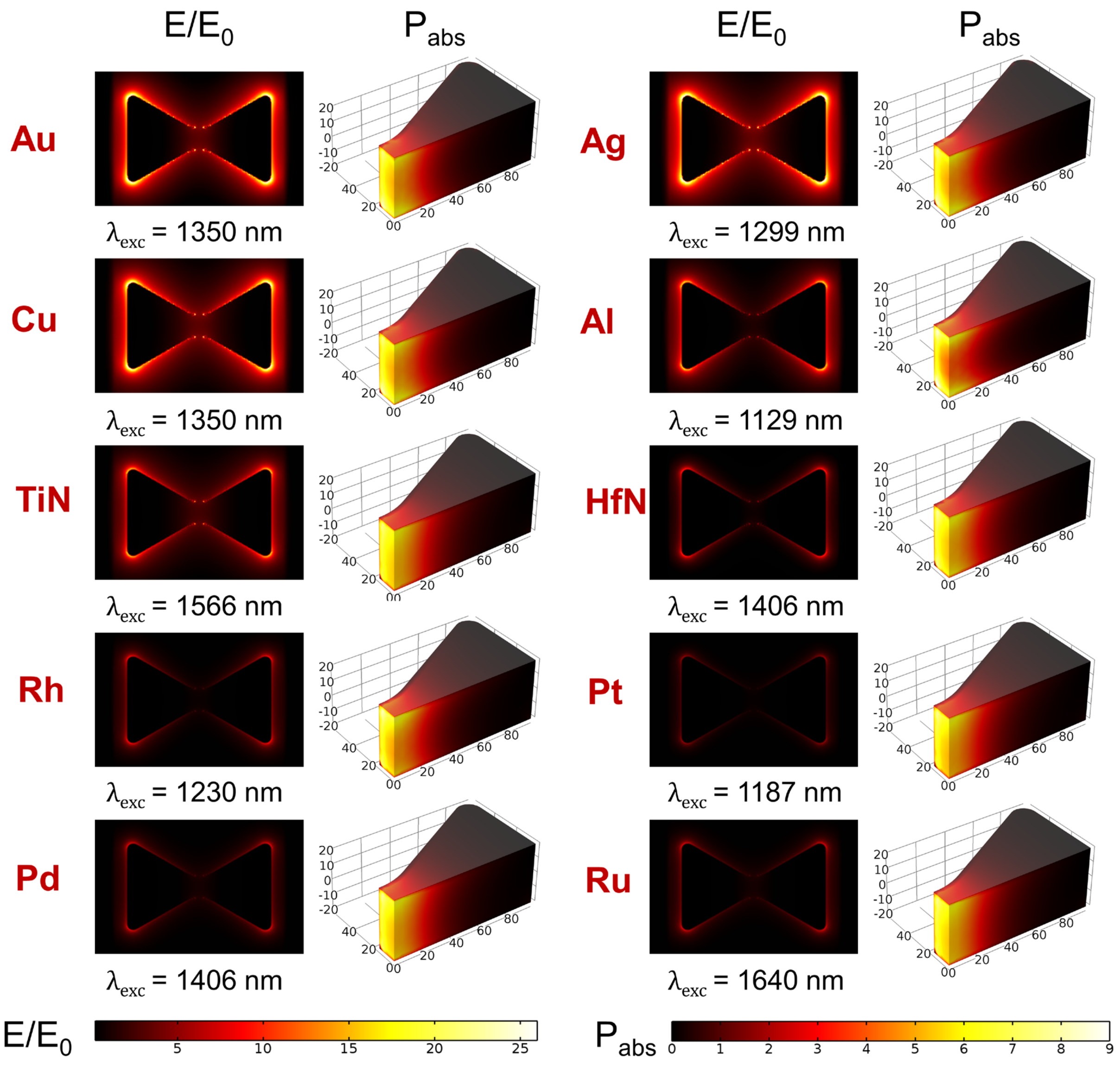

Figure S14. Simulated electric field maps (at top of structure) and normalized spatial absorbed power maps for nanoreactors (on Al_2O_3 substrate and in air surroundings) made from Au, Ag, Cu, Al, TiN, HfN, Rh, Pt, Pd, and Ru at 100% scale. Simulations were performed with plane wave excitation polarized along the long axis of the diabolo and at their respective peak absorption wavelength, as designated underneath each E-field map. Axes show dimensions in nm. The data shows that noble metals show the highest field electric field enhancement. It is also clear that the normalized absorbed power map changes only marginally between different metals.

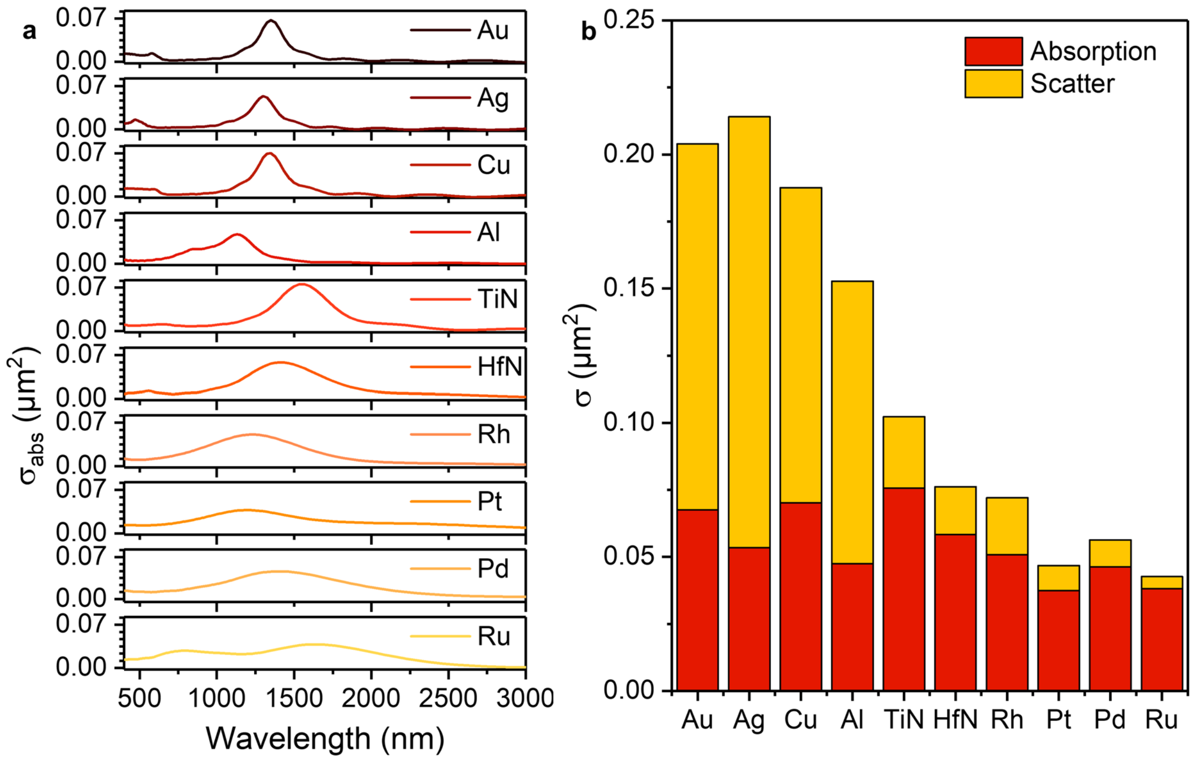

Figure S15. FDTD-simulated absorption cross section spectrum (a) and peak absorption and scatter cross sections (b) for metallic nanoreactors at 100% scale, made from Au, Ag, Cu, Al, TiN, HfN, Rh, Pt, Pd, and Ru. For reference: the area of the diabolo top surface is 0.014 μm^2. Optical constants were used from Johnson and Christy (Au),[27] CRC Handbook (Cu and Ag),[38] and Palik (Al, Rh, Pt, Pd, and Ru),[39] and from high quality TiN and HfN thin films.[26,40]

Table S3. Simulation parameters for other metals. The *G*-value for Rh was estimated according to Equation 14, with $\lambda = 0.377$, $\langle \omega_2 \rangle = 1.334 \times 10^{-41}$ J^2, and $M_W = 0.1029$ kg/mol.[41] Most of the thermal conductivity and heat capacity functions were taken from the COMSOL materials database ("Built-in"), except for TiN.

**Material**	**Al**	**Cu**	**Ag**	**Ru**	**Pd**	**Pt**	**Rh**	**TiN**
γ (10^{-3} J/mol.K^2)	1.35 [10]	0.695 [18]	0.646 [10]	3.35 [42]	9.9 [42]	6.63 [42]	4.89 [42]	3 [2,43]
a	0.01	0.01	0.01	0.01	0.01	0.01	0.01	0.25
b	0.99	0.99	0.99	0.99	0.99	0.99	0.99	0.75
ρ (kg/m^3)	2700	8940	10500	12450	11998	21388	12400	5210
G (10^{17} W/m^3.K)	2.45 [44]	1 [23]	0.35 [44]	11 [23]	5 [45]	2.5 [23]	19.5 (calc.)	35 [11]
$\kappa(T)$	Built-in	Built-in	Built-in	Built-in	Built-in	Built-in	Built-in	Lit. [43]
$C_l(T_l)$	Built-in	Built-in	Built-in	Built-in	Built-in	Built-in	Built-in	Lit. [43]

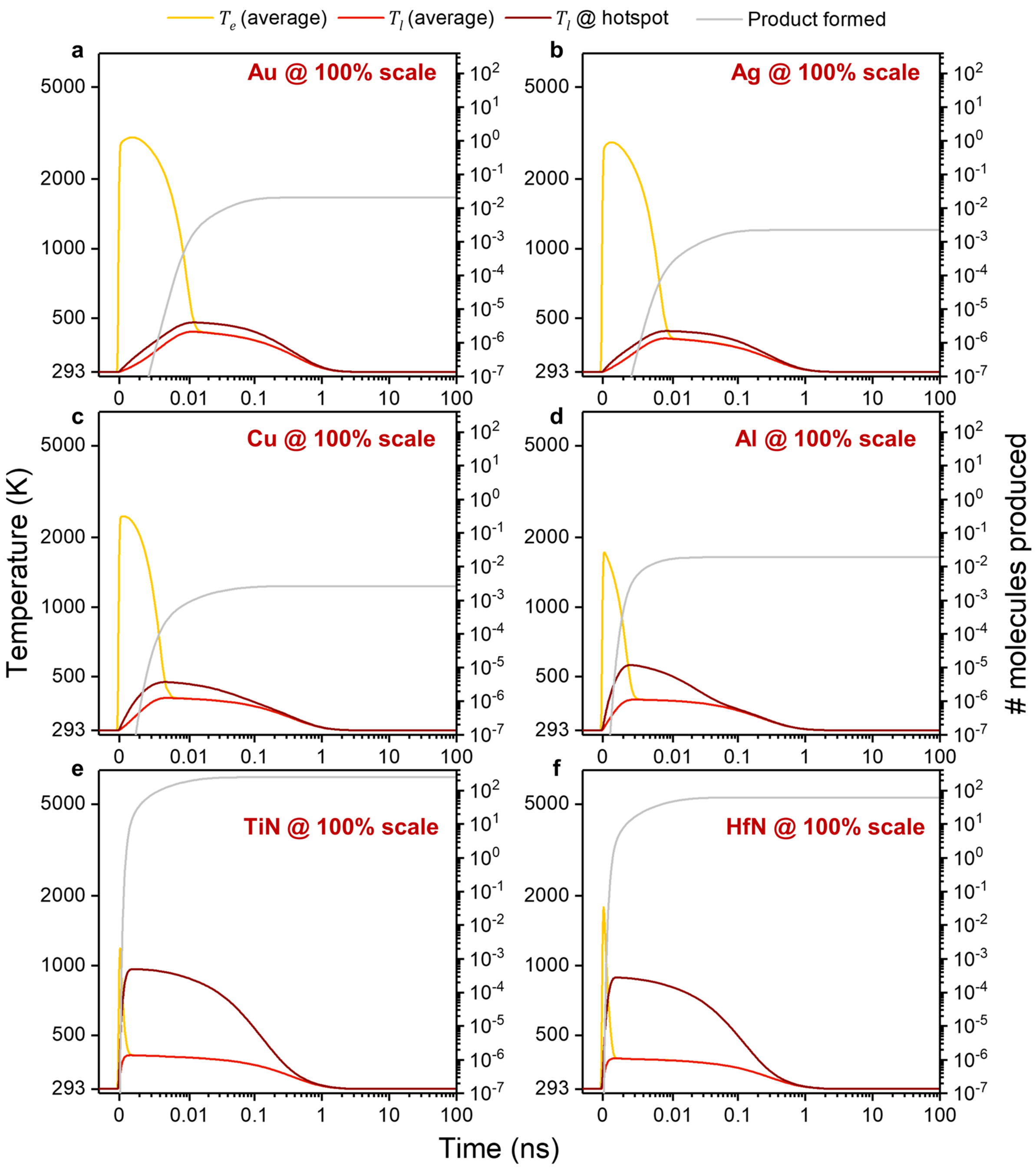

Figure S16 (continues on next page).

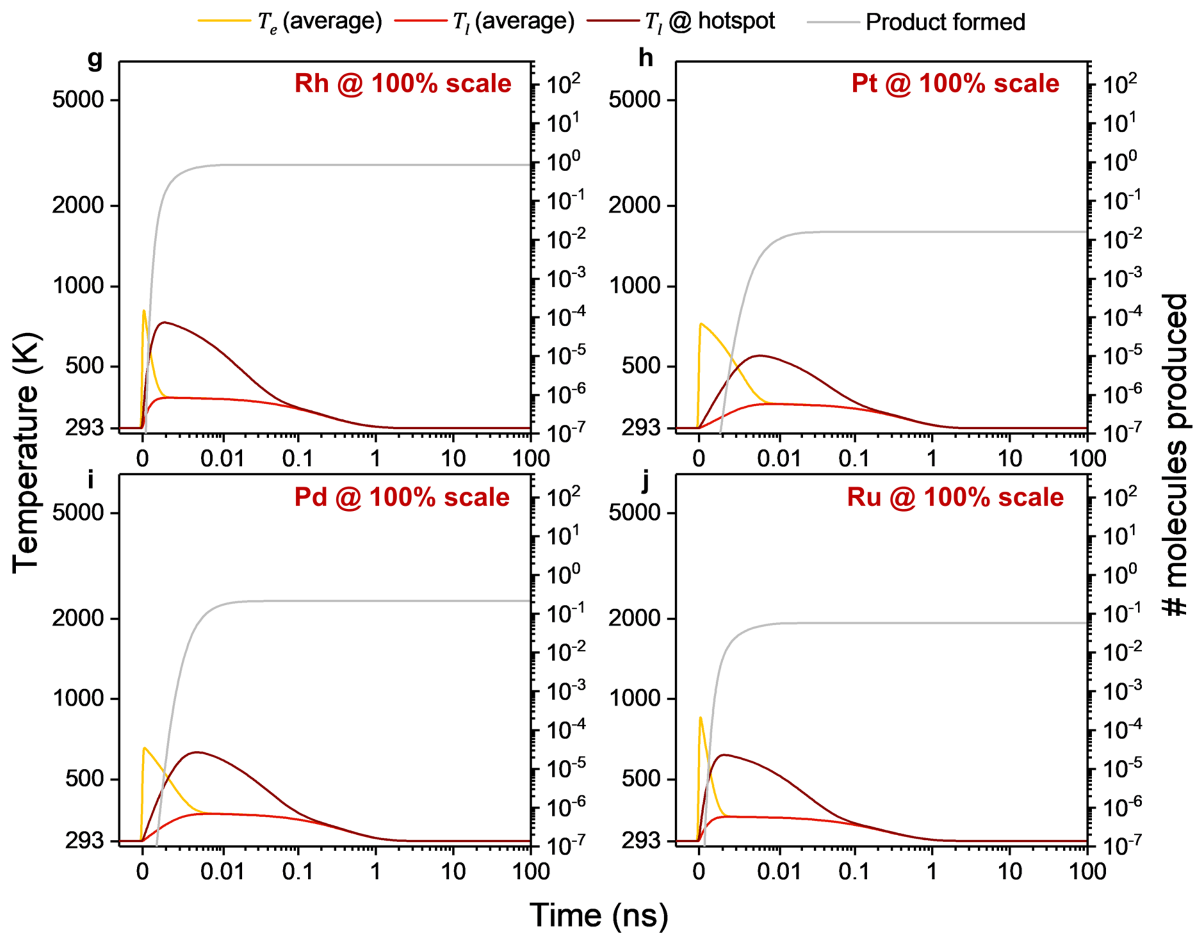

Figure S16 (continued). Time-evolution of volume-averaged electronic (yellow) and lattice temperatures (red), the lattice temperature at the absorption hotspot (dark red), and the number of product molecules produced per pulse (grey) during 50 fs pulsed excitation at 0.32 mJ/cm^2 pulse energy (0.8 W/cm^2) for Au (a), Ag (b), Cu (c), Al (d), TiN (e), HfN (f), Rh (g), Pt (h), Pd (i), and Ru (j) for nanoreactors on Al_2O_3 substrate and in air, at 100% scale. For these simulations, the specific material parameters used are listed in Table S3.

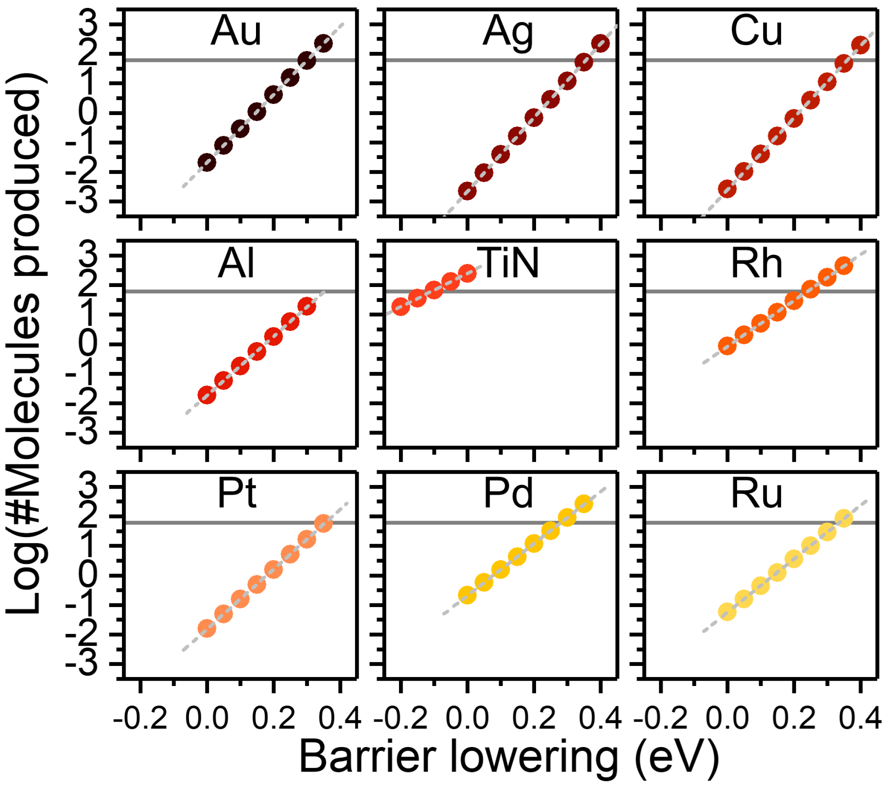

Figure S17. Chemical reaction yield for Au, Ag, Cu, Al, TiN, Rh, Pt, Pd, and Ru nanoreactors, driven at their respective maximum wavelength and corresponding absorption cross section, and as a function of activation energy (see Equation 21). Each point represents an individual simulation. Here, 0 barrier lowering corresponds to an activation energy of 1 eV (96 kJ/mol). The grey dashed lines are linear fits to the data, and the black lines indicate the performance of the HfN nanoreactor (62 molecules produced per pulse). From the intersection of both lines, the barrier lowering was calculated to achieve the same amount of chemical product. Simulations were performed with 50 fs pulsed excitation at 0.32 mJ/cm^2 pulse energy (0.8 W/cm^2), at 100% nanoreactor scale.

6. FDTD and heat transfer modelling for a nanosphere dimer

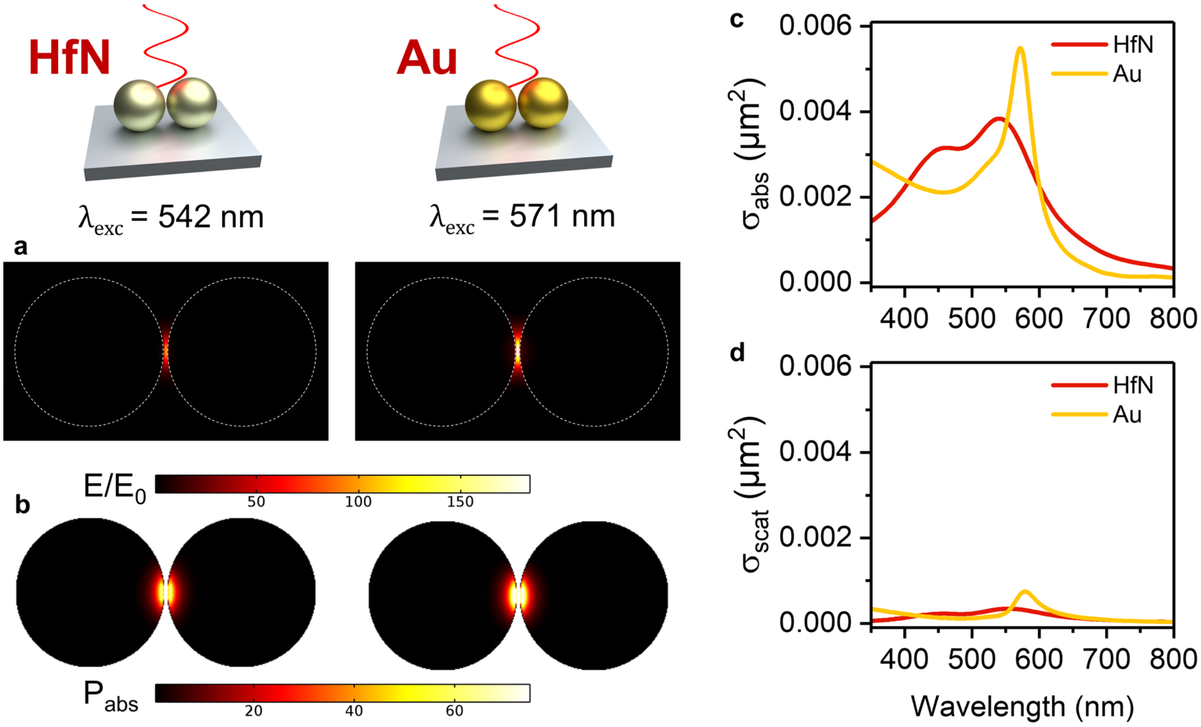

Figure S18. Optical simulations of metallic nanosphere dimers. a&b) FDTD-simulated electric field maps (at center of structure, a) and normalized spatial absorbed power maps (b) in HfN (left) and Au (right) nanosphere dimers in air and on Al_2O_3 substrate. The dimer consists of two 40 nm diameter spheres with an interparticle spacing of 1 nm. Simulations were performed with plane wave excitation polarized along the long axis of the dimer (x-axis) and at their respective peak absorption wavelength, as designated above each E-field map. c&d) Absorption (c) and scatter (d) cross section spectra for Au (yellow) and HfN (red) nanosphere dimers.

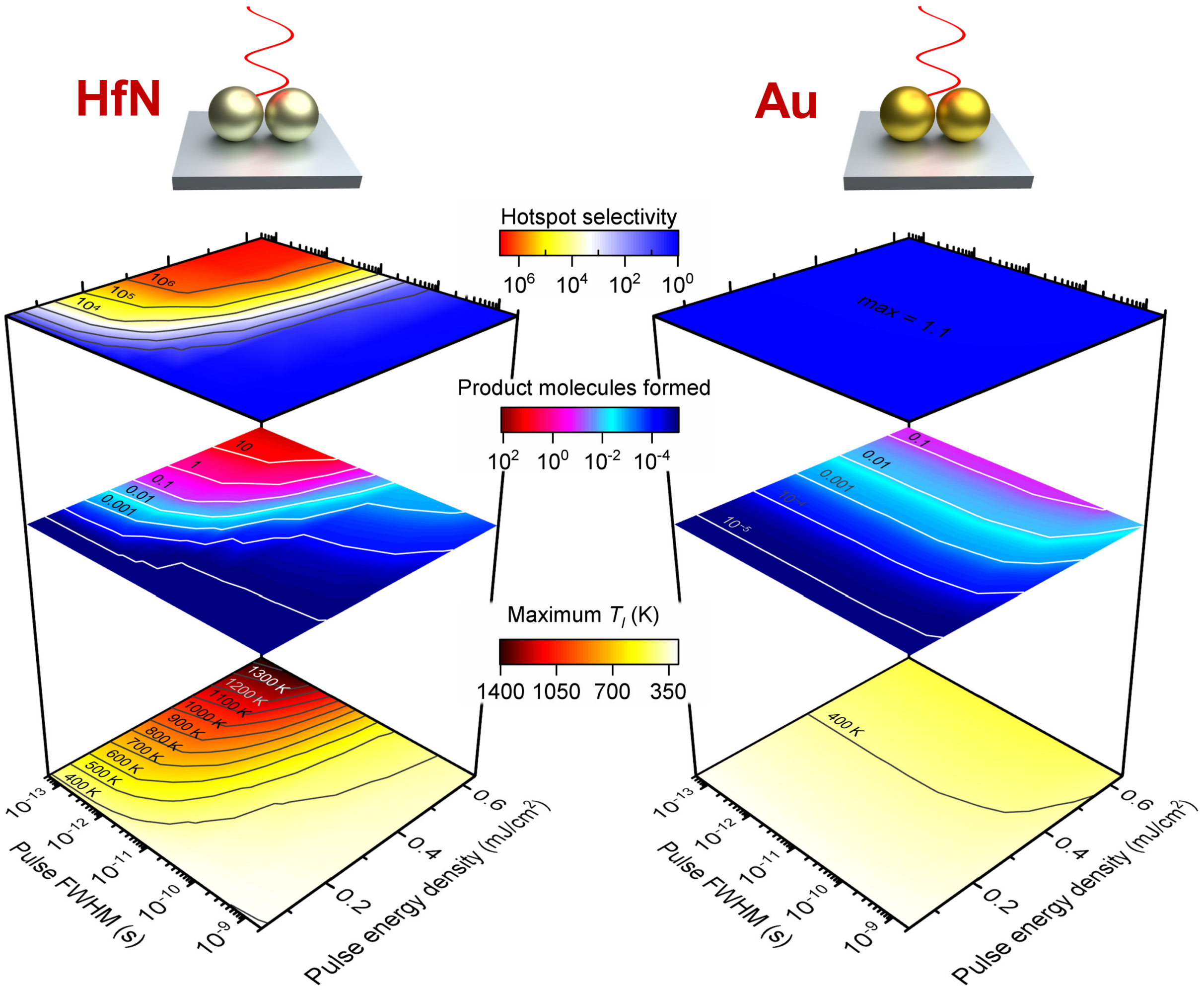

Figure S19. Influence of laser pulse duration (Gaussian time profile, FWHM = Full Width at Half Maximum) and pulse energy density on the maximum attained lattice temperature (bottom), amount of product molecules formed per pulse (middle), and the center selectivity of the chemical product formation (top) for HfN (left) and Au (right) nanosphere dimer reactors. A total of 48 simulations were used to obtain the contour plots. The center selectivity is defined as the ratio of product formed at the hotspot *and* the product formed at the point furthest away from the hotspot. Reactant depletion does not occur: the maximum reactant conversion is 9% for HfN at 0.64 mJ/cm^2 (1.6 W/cm^2) and 50 fs FWHM. Note that there is no transient thermal hotspot for the Au dimer under any irradiation conditions, therefore resulting in no hotspot selectivity, <1000× lower conversion, and drastically lower maximum surface T_l.

7. References

1. Zhang, Y., Tzou, D. Y. & Chen, J. K. Micro-and nanoscale heat transfer in femtosecond laser processing of metals. in *High-Power and Femtosecond Lasers: Properties, Materials and Applications* 159–206 (Nova Science Publishers, Inc., 2009).

2. George, H. *et al.* Nonlinearities and carrier dynamics in refractory plasmonic TiN thin films. *Opt. Mater. Express* **9**, 3911 (2019).

3. COMSOL. *Heat Transfer Module User Guide v5.4, Chapter 4, Theory for the Local Thermal Non-Equilibrium Interface*. (2018). https://doc.comsol.com/5.4/doc/com.comsol.help.heat/HeatTransferModuleUsersGuide.pdf.

4. Baffou, G. & Rigneault, H. Femtosecond-pulsed optical heating of gold nanoparticles. *Phys. Rev. B* **84**, 035415 (2011).

5. Gundrum, B. C., Cahill, D. G. & Averback, R. S. Thermal conductance of metal-metal interfaces. *Phys. Rev. B* **72**, 245426 (2005).

6. Costescu, R. M., Wall, M. A. & Cahill, D. G. Thermal conductance of epitaxial interfaces. *Phys. Rev. B* **67**, 054302 (2003).

7. Metwally, K., Mensah, S. & Baffou, G. Fluence Threshold for Photothermal Bubble Generation Using Plasmonic Nanoparticles. *J. Phys. Chem. C* **119**, 28586–28596 (2015).

8. Hashimoto, S., Werner, D. & Uwada, T. Studies on the interaction of pulsed lasers with plasmonic gold nanoparticles toward light manipulation, heat management, and nanofabrication. *J. Photochem. Photobiol. C Photochem. Rev.* **13**, 28–54 (2012).

9. Westrum, E. F. & Sommers, J. A. Heat capacity of hafnium mononitride from temperatures of 5 to 350 K. An estimation procedure. *J. Therm. Anal. Calorim.* **69**, 103–112 (2002).

10. Ashcroft, N. W.; Mermin, N. D. *Solid state physics*. (New York : Holt, Rinehart and Winston, 1978).

11. Dal Forno, S. & Lischner, J. Electron-phonon coupling and hot electron thermalization in titanium nitride. *Phys. Rev. Mater.* **3**, 115203 (2019).

12. Saha, B., Acharya, J., Sands, T. D. & Waghmare, U. V. Electronic structure, phonons, and thermal properties of ScN, ZrN, and HfN: A first-principles study. *J. Appl. Phys.* **107**, 033715 (2010).

13. Gupta, S. D., Gupta, S. K. & Jha, P. K. High pressure study on the phonon spectra and thermal properties in hafnium nitride and zirconium nitride. *J. Therm. Anal. Calorim.* **107**, 49–53 (2012).

14. Pierson, H. O. Carbides of Group VI. in *Handbook of Refractory Carbides and Nitrides* 100–117 (Elsevier, 1996). doi:10.1016/B978-081551392-6.50007-6.

15. Opeka, M. M., Talmy, I. G., Wuchina, E. J., Zaykoski, J. A. & Causey, S. J. Mechanical, Thermal, and Oxidation Properties of Refractory Hafnium and zirconium Compounds. *J. Eur. Ceram. Soc.* **19**, 2405–2414 (1999).

16. Wuchina, E. *et al.* Designing for ultrahigh-temperature applications: The mechanical and

thermal properties of HfB2, HfCx, HfNx and αHf(N). *J. Mater. Sci.* **39**, 5939–5949 (2004).

17. Li, S. *et al.* Anomalous thermal transport in metallic transition-metal nitrides originated from strong electron–phonon interactions. *Mater. Today Phys.* **15**, 100256 (2020).

18. Kittel, C. *Introduction to Solid State Physics, 8th edition*. *Wiley & Sons* (2004).

19. Allen, P. B. Theory of thermal relaxation of electrons in metals. *Phys. Rev. Lett.* **59**, 1460–1463 (1987).

20. Chen, X. J. *et al.* Pressure-induced phonon frequency shifts in transition-metal nitrides. *Phys. Rev. B - Condens. Matter Mater. Phys.* **70**, 014501 (2004).

21. Chauhan, M. & Gupta, D. C. Structural, electronic, mechanical and thermo-physical properties of TMN (TM = Ti, Zr and Hf) under high pressures: A first-principle study. *Int. J. Refract. Met. Hard Mater.* **42**, 77–90 (2014).

22. Spengler, W., Kaiser, R., Christensen, A. N. & Müller-Vogt, G. Raman scattering, superconductivity, and phonon density of states of stoichiometric and nonstoichiometric TiN. *Phys. Rev. B* **17**, 1095–1101 (1978).

23. Hohlfeld, J. *et al.* Electron and lattice dynamics following optical excitation of metals. *Chem. Phys.* **251**, 237–258 (2000).

24. Diroll, B. T., Saha, S., Shalaev, V. M., Boltasseva, A. & Schaller, R. D. Broadband Ultrafast Dynamics of Refractory Metals: TiN and ZrN. *Adv. Opt. Mater.* **8**, 2000652 (2020).

25. Brorson, S. D. *et al.* Femtosecond room-temperature measurement of the electron-phonon coupling constant in metallic superconductors. *Phys. Rev. Lett.* **64**, 2172–2175 (1990).

26. Askes, S. H. C., Schilder, N. J., Zoethout, E., Polman, A. & Garnett, E. C. Tunable plasmonic HfN nanoparticles and arrays. *Nanoscale* **11**, 20252–20260 (2019).

27. Johnson, P. B. & Christy, R. W. Optical Constants of the Noble Metals. *Phys. Rev. B* **6**, 4370–4379 (1972).

28. Davis, M. E. & Davis, R. J. *Fundamentals of Chemical Reaction Engineering*. *McGraw-Hill* (McGraw-Hill, 2013).

29. Criado, J. M., Pérez-Maqueda, L. A. & Sánchez-Jiménez, P. E. Dependence of the preexponential factor on temperature: Errors in the activation energies calculated by assuming that A is constant. *J. Therm. Anal. Calorim.* **82**, 671–675 (2005).

30. Habib, A., Florio, F. & Sundararaman, R. Hot carrier dynamics in plasmonic transition metal nitrides. *J. Opt.* **20**, 064001 (2018).

31. Zhang, Y. *et al.* Surface-Plasmon-Driven Hot Electron Photochemistry. *Chem. Rev.* **118**, 2927–2954 (2018).

32. Yang, W., Liu, Y., McBride, J. R. & Lian, T. Ultrafast and Long-Lived Transient Heating of Surface Adsorbates on Plasmonic Semiconductor Nanocrystals. *Nano Lett.* **21**, 453–461 (2021).

33. Öberg, H. *et al.* Optical laser-induced CO desorption from Ru(0001) monitored with a

free-electron X-ray laser: DFT prediction and X-ray confirmation of a precursor state. *Surf. Sci.* **640**, 80–88 (2015).

34. Nilsson, A. *et al.* Catalysis in real time using X-ray lasers. *Chem. Phys. Lett.* **675**, 145–173 (2017).

35. Schlögl, R. Heterogeneous catalysis. *Angew. Chemie - Int. Ed.* **54**, 3465–3520 (2015).

36. Andersen, M., Panosetti, C. & Reuter, K. A practical guide to surface kinetic Monte Carlo simulations. *Front. Chem.* **7**, 1–24 (2019).

37. Jain, P. K., Qian, W. & El-Sayed, M. A. Ultrafast cooling of photoexcited electrons in gold nanoparticle-thiolated DNA conjugates involves the dissociation of the gold-thiol bond. *J. Am. Chem. Soc.* **128**, 2426–2433 (2006).

38. Haynes, W. M. *Handbook of Chemistry and Physics*. (CRC Press, 2017).

39. Palik, E. D. *Handbook of optical constants of solids*. *Handbook of Optical Constants of Solids* (2012). doi:10.1016/C2009-0-20920-2.

40. Briggs, J. A. *et al.* Temperature-dependent optical properties of titanium nitride. *Appl. Phys. Lett.* **110**, 101901 (2017).

41. Bose, S. K. Electron-phonon coupling and spin fluctuations in 3d and 4d transition metals: Implications for superconductivity and its pressure dependence. *J. Phys. Condens. Matter* **21**, (2009).

42. Gopal, E. S. R. Electronic Specific Heat. in *Specific Heats at Low Temperatures* 55–83 (Springer US, 1966). doi:10.1007/978-1-4684-9081-7_4.

43. Doiron, B. *et al.* Optimizing hot electron harvesting at planar metal-semiconductor interfaces with titanium oxynitride thin films. 1–21 (2018) doi:arXiv:1807.03702.

44. Lin, Z., Zhigilei, L. V. & Celli, V. Electron-phonon coupling and electron heat capacity of metals under conditions of strong electron-phonon nonequilibrium. *Phys. Rev. B - Condens. Matter Mater. Phys.* **77**, 1–17 (2008).

45. Wang, L., Sagaguchi, T., Okuhata, T., Tsuboi, M. & Tamai, N. Electron and Phonon Dynamics in Hexagonal Pd Nanosheets and Ag/Pd/Ag Sandwich Nanoplates. *ACS Nano* **11**, 1180–1188 (2017).
